# S1PR1 inhibition induces proapoptotic signaling in T cells and limits humoral responses within lymph nodes

**DOI:** 10.1172/JCI174984

**Published:** 2024-01-09

**Authors:** Dhaval Dixit, Victoria M. Hallisey, Ethan Y.S. Zhu, Martyna Okuniewska, Ken Cadwell, Jerry E. Chipuk, Jordan E. Axelrad, Susan R. Schwab

**Affiliations:** 1 Departments of Cell Biology and Pathology, New York University Grossman School of Medicine, New York, New York, USA.; 2 Department of Medicine and Institute for Immunology, University of Pennsylvania Perelman School of Medicine, Philadelphia, Pennsylvania, USA.; 3 Department of Oncological Sciences, Department of Dermatology, and Tisch Cancer Institute, Icahn School of Medicine at Mount Sinai, New York, New York, USA.; 4 Division of Gastroenterology, Department of Medicine, New York University Grossman School of Medicine, New York, New York, USA.

**Keywords:** Immunology, Adaptive immunity, Apoptosis, Cell migration/adhesion

## Abstract

Effective immunity requires a large, diverse naive T cell repertoire circulating among lymphoid organs in search of antigen. Sphingosine 1-phosphate (S1P) and its receptor S1PR1 contribute by both directing T cell migration and supporting T cell survival. Here, we addressed how S1P enables T cell survival and the implications for patients treated with S1PR1 antagonists. We found that S1PR1 limited apoptosis by maintaining the appropriate balance of BCL2 family members via restraint of JNK activity. Interestingly, the same residues of S1PR1 that enable receptor internalization were required to prevent this proapoptotic cascade. Findings in mice were recapitulated in ulcerative colitis patients treated with the S1PR1 antagonist ozanimod, and the loss of naive T cells limited B cell responses. Our findings highlighted an effect of S1PR1 antagonists on the ability to mount immune responses within lymph nodes, beyond their effect on lymph node egress, and suggested both limitations and additional uses of this important class of drugs.

## Abstract 2

<p>Sphingosine 1-phosphate receptor 1 inhibition induces a pro-apoptotic cascade in murine and human T cells, and limits initiation of appropriate immune responses within lymph nodes.</p>

## Graphical abstract

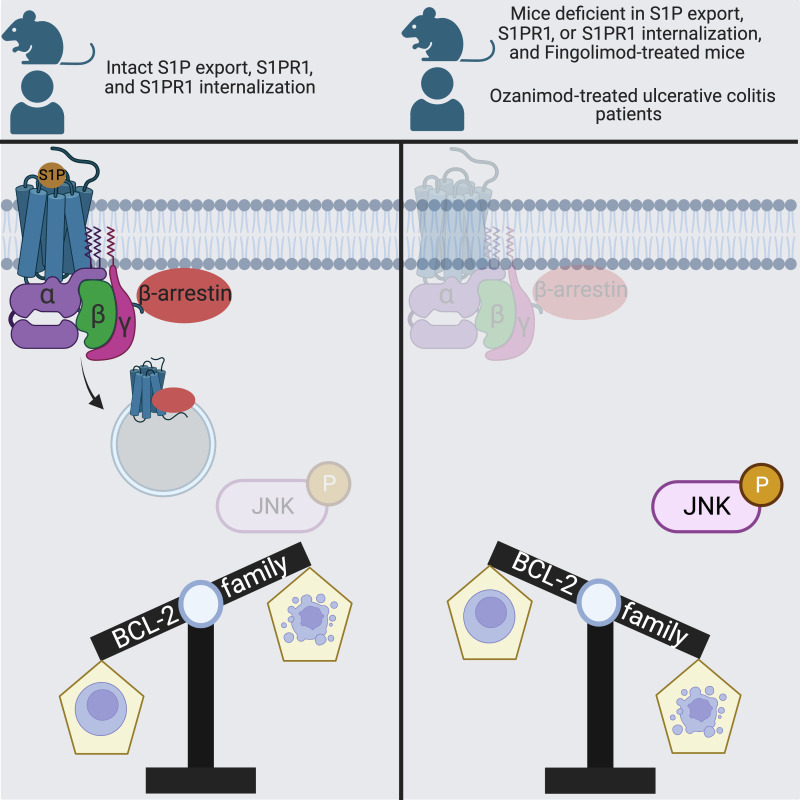

## Introduction

Regulated cell death is an integral process in mammalian development and homeostasis. T cells are often described as “primed” for apoptosis; developing T cells with non-functional or self-reactive T cell receptors must be eliminated (1, 2), and the bulk of effector T cells in an immune response must die to prevent excessive inflammation (3). However, naive T cells must be long-lived to maintain a diverse repertoire to defend against diverse pathogens, and memory T cells must be long-lived to confer lasting protection (4, 5). A T cell makes critical decisions about whether to live or die at each stage of its life, and the factors that the cell weighs remain incompletely understood.

The signaling lipid sphingosine 1-phosphate (S1P) has been intensively studied in the context of T cell migration (6). The high concentration of S1P in blood and lymph guides T cells out of the low-S1P environment of lymphoid organs, and T cells follow this gradient primarily using S1P receptor 1 (S1PR1). Without circulatory S1P or without S1PR1 on T cells, T cells fail to exit the thymus into blood, and fail to exit lymph nodes (LNs) into lymph. We have found that S1P, acting via S1PR1, also plays an essential role in naive T cell survival, which, interestingly, is independent of its role in cell migration (7).

Four drugs that target S1PR1 have been approved by the FDA to treat multiple sclerosis, and two have been approved to treat ulcerative colitis. These drugs work in part by blocking pathogenic T cells from exiting LNs, thus preventing them from accessing the brain or colon (8, 9). However, some effects of these drugs are unexplained. Patients treated with drugs targeting S1PR1 respond poorly to SARS-CoV-2 vaccines (10–12), although B cell responses should be initiated within LNs. Moreover, antibody titers have been reported to correlate negatively with time on drug, while the drugs’ effects on cell migration occur within hours of the first dose and rapidly reach a new equilibrium (10–12). Finally, the few studies measuring blood lymphocyte counts in patients who have stopped treatment suggest that some patients do not fully recover cell numbers (13, 14).

We know little about how S1P supports T cell survival, or about how the effects of genetic blockade of S1P signaling in mice translate to the many patients treated with drugs targeting S1PR1. Here we addressed these questions. We found that S1PR1 activation restricted JNK phosphorylation, thereby maintaining the appropriate balance of BCL2 family members within the T cell, and in turn limiting apoptosis. Interestingly, the same residues of the S1PR1 C-terminus that enable receptor internalization were required to prevent the apoptotic cascade. Findings using genetic models were recapitulated in mice treated with the drug fingolimod (FTY720), which targets S1PR1, and in ulcerative colitis patients treated with the drug ozanimod, also an S1PR1 modulator. Using a mouse model, we found that poor antibody responses after prolonged treatment with S1PR1 antagonists may in part reflect loss of the naive T cell repertoire, pointing to an important role of S1P signaling in regulating T cell responses within the LNs as well as in non-lymphoid tissues. Our findings simultaneously suggest reasons for caution in use of S1PR1 antagonists and additional uses for these drugs.

## Results

### The S1P transporter SPNS2 and S1PR1 prevent naive T cell death.

**Figure 1:**
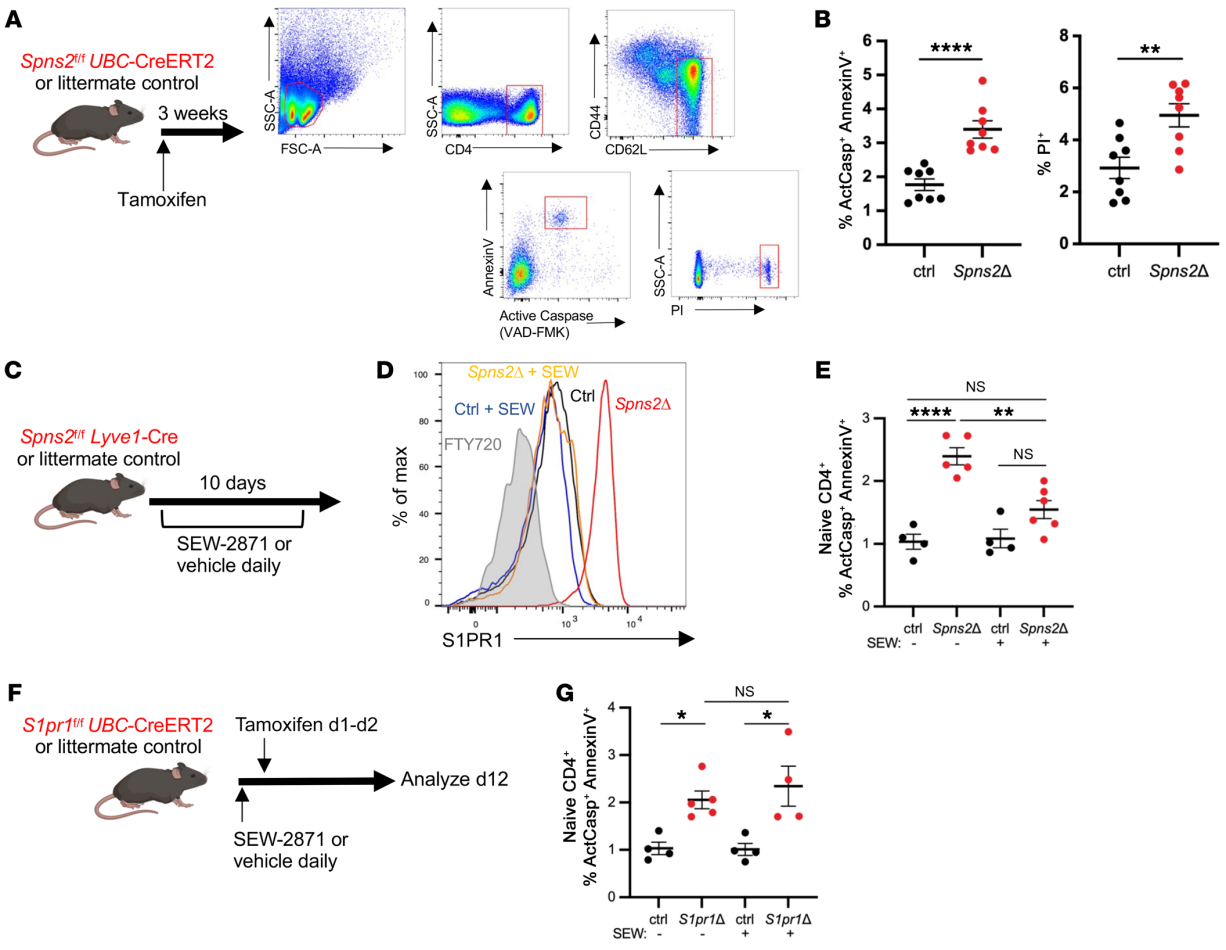
SPNS2-derived S1P and S1PR1 prevent apoptotic death of naive T cells. (**A** and **B**): (**A**) *Spns2^fl/fl^* *UBC*-CreERT2 mice and littermate controls were treated with tamoxifen, and 3–4 weeks later LN T cells were analyzed by flow cytometry. (**B**) Frequency of active caspase–positive (ActCasp^+^) annexin V^+^ and propidium iodide–positive (PI^+^) cells among naive CD4^+^ T cells. Compilation of 4 experiments, 8 mice per group. (**C**–**E**): (**C**) *Spns2^fl/fl^* *Lyve1*-Cre and littermate controls were treated with 10 mg/kg SEW-2871 or vehicle daily. After 10 days of treatment, LN T cells were analyzed by flow cytometry. (**D**) Representative histogram of S1PR1 expression on naive CD4^+^ T cells from LNs of *Spns2Δ* mice and littermate controls, with and without SEW-2871 treatment. (**E**) Frequency of ActCasp^+^ annexin V^+^ cells among naive CD4^+^ T cells in LNs of *Spns2Δ* mice and littermate controls, with and without SEW-2871 treatment. Compilation of 4 experiments, 4–6 mice per group. (**F** and **G**): (**F**) *S1pr1^fl/fl^* *UBC*-CreERT2 mice and littermate controls were treated with 10 mg/kg SEW-2871 or vehicle daily for 12 days and treated with tamoxifen on day 1 and day 2. On day 12, LN cells were analyzed by flow cytometry. (**G**) Frequency of ActCasp^+^ annexin V^+^ cells among naive CD4^+^ T cells in LNs of *S1pr1Δ* mice and littermate controls, with and without SEW-2871 treatment. Compilation of 4 experiments with *n* = 4–5 mice per group. **B**, Student’s *t* test; **E** and **G**, 1-way ANOVA with multiple comparisons. *P ≤ 0.05, **P ≤ 0.01, ****P ≤ 0.0001.

We began by further testing the role of S1P in promoting naive T cell survival. We had previously shown that in mice lacking the S1P transporter SPNS2 in *Lyve1*-expressing cells, which include lymphatic endothelial cells (*Spns2^fl/fl^ Lyve1-*Cre, *Spns2Δ^Lyve1^*), lymph S1P was lost (Supplemental Figure 1A; supplemental material available online with this article; https://doi.org/10.1172/JCI174984DS1) (7). As expected, naive T cells were retained in LNs because there was no lymph S1P to guide them out, and naive T cells were no longer exposed to circulatory S1P (7). Surprisingly, they died at an increased rate compared with T cells in LNs of littermate controls (7). To assess potential developmental defects contributing to T cell death, we deleted *Spns2* in adults. We bred *Spns2^fl/fl^ UBC*-CreERT2 mice, with widespread expression of tamoxifen-inducible Cre (15), and treated adults with tamoxifen 3 weeks before analysis (tamoxifen-treated *Spns2^fl/fl^ UBC*-CreERT2 mice, *Spns2Δ^UBC^*). We measured cell death by flow cytometry, assessing propidium iodide uptake as well as staining with a probe for active caspases and with annexin V (Figure 1A). We found an increased percentage of dying cells among naive T cells in *Spns2Δ^UBC^* LNs compared with tamoxifen-treated littermate controls, as well as a loss of naive T cells (Figure 1B and Supplemental Figure 1, B–D). Consistent with the importance of S1P secretion by SPNS2, we found an increased percentage of dying cells among naive T cells in LNs of mice with *Lyve1*-Cre–mediated deletion of the 2 sphingosine kinases that synthesize S1P (*Sphk1^fl/fl^ Sphk2^–/–^ Lyve1-*Cre, *SphkΔ^Lyve1^*) (16) (Supplemental Figure 1E). Finally, we confirmed previous findings that S1PR1 was required in a cell-intrinsic manner to limit naive T cell death, using mixed bone marrow chimeras and transfer experiments (ref. 7, and see below). These data suggest a model in which S1P secreted via SPNS2 and sensed by T cells via S1PR1 promotes naive T survival.

### S1P signaling promotes naive T cell survival independent of exit from LNs or uptake of S1P.

S1P could promote naive T cell survival by at least 3 possible mechanisms: enabling T cell trafficking among or within lymphoid organs, acting as a metabolite or protein cofactor after uptake into the T cell, or activating a signaling pathway downstream of S1PR1 within the T cell.

Our previous work had indicated that S1PR1 did not promote survival by enabling exit from LNs or access to IL-7 or peptide-MHC within LNs (ref. 7, and see below). We next considered the hypothesis that S1P uptake was important for T cell survival. S1PR1 is a G protein–coupled receptor (GPCR), and, like many GPCRs, S1PR1 is internalized after binding its ligand (17, 18). Intracellular S1P has been implicated in many processes important for cell survival, from acting as a metabolic intermediate to promoting mitochondrial homeostasis to regulating glycolysis (19–22). To test the role of S1P uptake by S1PR1 in T cell survival, we took advantage of a chemical agonist of S1PR1, SEW-2871, which is structurally distinct from S1P (23). We treated *Spns2Δ^Lyve1^* mice and littermate controls with SEW-2871 or vehicle daily for 10 days (Figure 1C). Naive T cells in both *Spns2Δ^Lyve1^* mice and controls treated with SEW-2871 expressed lower levels of surface S1PR1 than naive T cells in vehicle-treated mice (Figure 1D), consistent with previous findings that SEW-2871 bound the receptor and induced its internalization. As expected, in *Spns2Δ^Lyve1^* mice, the increased S1PR1 signal in LNs did not restore egress. As expected, in control mice, increased agonism of S1PR1 in LNs disrupted the ligand gradient that would normally guide exit from LNs (normally low S1PR1 agonism in LNs, now elevated due to the presence of SEW-2871; normally high S1PR1 agonism in lymph, still high with the continued presence of S1P) (Supplemental Figure 1F). We found that SEW-2871–treated *Spns2Δ^Lyve1^* mice had a reduced frequency of dying naive T cells compared with vehicle-treated *Spns2Δ^Lyve1^* mice (Figure 1E and Supplemental Figure 1, G and H). This result suggested that signaling through S1PR1, rather than S1P uptake (or LN exit), was important for naive T cell survival. Importantly, SEW-2871 treatment of *S1pr1Δ^UBC^* mice did not rescue naive T cell apoptosis (Figure 1, F and G), indicating that SEW-2871 worked through S1PR1 to promote survival.

### Naive T cell apoptosis is not associated with changes in canonical signaling pathways downstream of S1PR1.

We then considered the possibility that S1PR1 activates a pro-survival signaling pathway within naive T cells. S1PR1 couples to Gα_i_ and its associated Gβγ subunits (24). Experiments using cultured cells have demonstrated that S1PR1, like many GPCRs, can activate AKT and ERK, kinases that promote survival in many contexts (25, 26). Furthermore, Gα_i_ inhibition of adenylate cyclase has been demonstrated in many cell types to prevent cAMP accumulation and hence limit protein kinase A (PKA) activation. Yet little is known about S1PR1-dependent signaling pathways in naive T cells in vivo. In LNs, T cells receive inputs from many other sources, including cytokine receptors, chemokine receptors, the T cell receptor, and metabolic sensors, and these also regulate AKT, ERK, and/or PKA signaling. We therefore asked whether S1PR1’s contribution was limiting in the context of the many other inputs into these pathways in a naive T cell in vivo.

**Figure 2:**
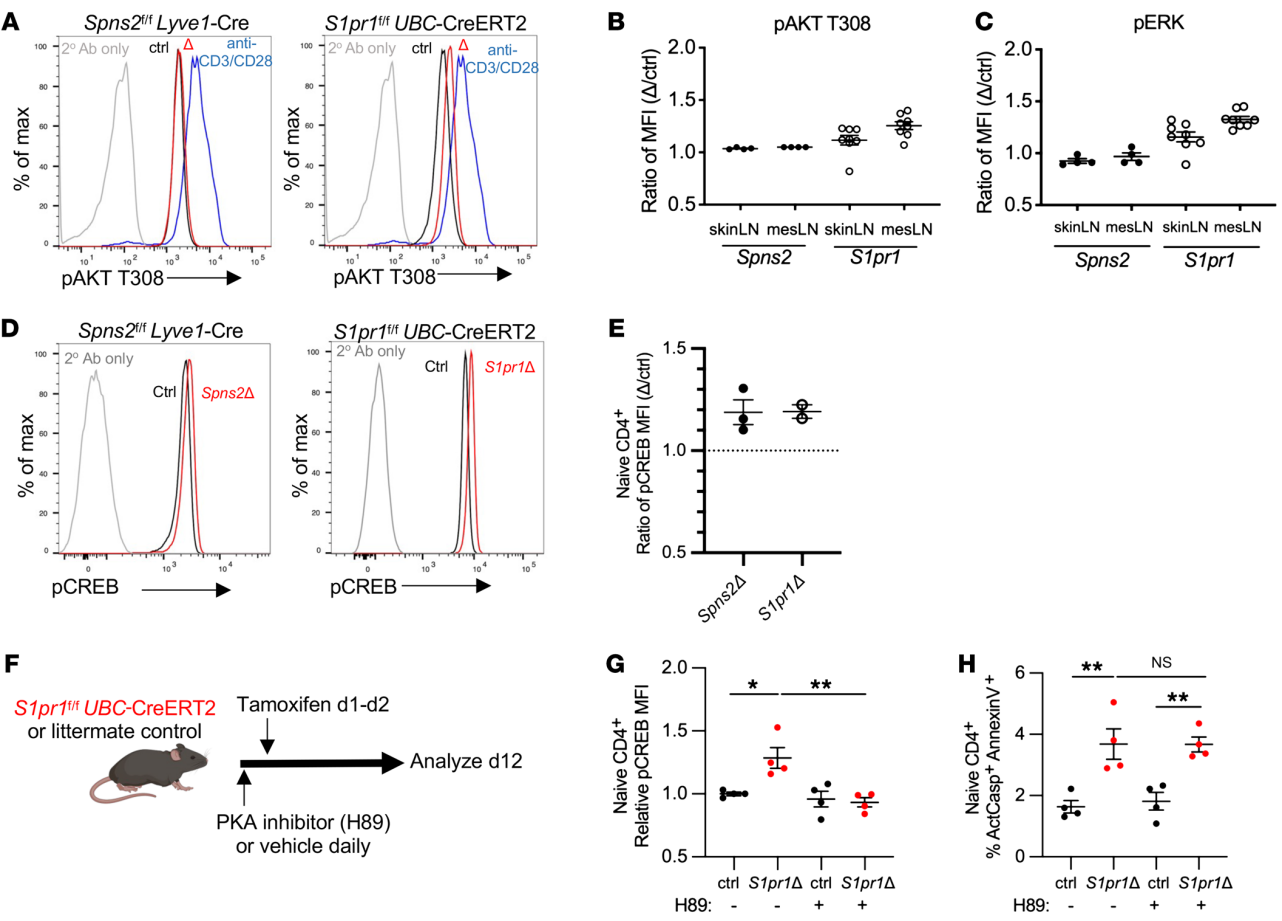
Canonical S1PR1 signaling pathways do not regulate naive T cell survival. (**A** and **B**) p-AKT T308 measured by flow cytometry. (**A**) Representative histogram of p-AKT T308 in naive CD4^+^ T cells from the skin-draining LNs of a *Spns2^fl/fl^ Lyve1*-Cre mouse and littermate control (left) or a *S1pr1^fl/fl^UBC*-CreERT2 mouse and littermate control (right) 3–4 weeks after tamoxifen treatment. p-AKT T308 in WT CD4^+^ T cells activated in vitro served as a comparison (same comparison used in both plots). (**B**) Compilation. Each point represents the ratio of the p-AKT T308 geometric mean fluorescence intensity (MFI) in naive CD4^+^ T cells from a *Spns2Δ* mouse to the p-AKT T308 MFI in naive CD4^+^ T cells from its littermate control, or the ratio of the p-AKT T308 MFI in naive CD4^+^ T cells from a *S1pr1Δ* mouse to the p-AKT T308 MFI in naive CD4^+^ T cells from its littermate control. SkinLN, skin-draining LNs; mesLN, mesenteric LNs. Compilation of 4 experiments with 4 pairs of mice for *Spns2* and 8 experiments with 8 pairs for *S1pr1*. (**C**) As in **B**, for p-ERK. (**D** and **E**) Phosphorylated CREB (p-CREB) measured by flow cytometry. (**D**) Representative histogram of p-CREB in naive CD4^+^ T cells from LNs of a *Spns2^fl/fl^* *Lyve1*-Cre mouse and littermate control (left) or a *S1pr1^fl/fl^* *UBC*-CreERT2 mouse and littermate control (right) 3–4 weeks after tamoxifen treatment. (**E**) As in **B**, for p-CREB. Compilation of 2–3 experiments with 2–3 per group. (**F**–**H**): (**F**) Experiment design. (**G**) Relative p-CREB expression in naive CD4^+^ T cells. Each point represents the MFI in 1 individual mouse divided by the mean MFI in vehicle-treated control mice in that experiment. (**H**) Frequency of ActCasp^+^ annexin V^+^ cells among naive CD4^+^ T cells in LNs. Compilation of 3 experiments, with 4 per group. **G** and **H**, 1-way ANOVA with multiple comparisons. *P ≤ 0.05, **P ≤ 0.01.

To study S1PR1’s contribution to AKT and/or ERK activation, we measured AKT and ERK phosphorylation in naive T cells from *Spns2Δ^Lyve1^* and *S1pr1Δ^UBC^* mice and littermate controls. We predicted that expression of phosphorylated AKT (p-AKT) or p-ERK would be decreased in naive T cells from *Spns2Δ^Lyve1^* and *S1pr1Δ^UBC^* mice if S1P signaling were a key input to the pathway. We detected no difference in p-AKT or p-ERK in naive T cells between *Spns2Δ^Lyve1^* mice and littermate controls. This could have simply reflected a limitation of the assay, but naive T cells from *S1pr1Δ^UBC^* mice had reproducibly increased levels of p-AKT and p-ERK compared with littermate controls (Figure 2, A–C). Since activity of these pathways was not decreased, we concluded that they were unlikely to account for S1P-dependent naive T cell survival. We were surprised by the divergence between *Spns2Δ* and *S1pr1Δ* T cells in p-AKT and p-ERK levels; we ultimately found that differential expression of the C-type lectin CD69 explained the difference (Supplemental Figure 2).

We also tested whether Gα_i_ inhibition of cAMP/PKA was important for S1P-dependent naive T cell survival. Activated PKA can phosphorylate the transcription factor CREB (27). We found that naive T cells from both *Spns2Δ* and *S1pr1Δ* mice had slightly increased phosphorylated CREB, suggesting that S1PR1’s input was limiting for inhibition of the cAMP/PKA/CREB pathway (Figure 2, D and E). To test whether this pathway was important in naive T cell survival, we treated *S1pr1Δ* mice daily with a PKA inhibitor, H-89 (28) (Figure 2F). Although the inhibitor reduced CREB phosphorylation, it did not affect the frequency of dying naive T cells, suggesting that a PKA-dependent pathway did not regulate S1P-dependent naive T cell survival (Figure 2, G and H).

### S1P signaling promotes survival by regulating the balance of BCL2 family proteins.

We next investigated whether loss of S1PR1 signaling resulted in changes in proximal regulators of cell death; we hoped to use this information to work back to the receptor.

**Figure 3:**
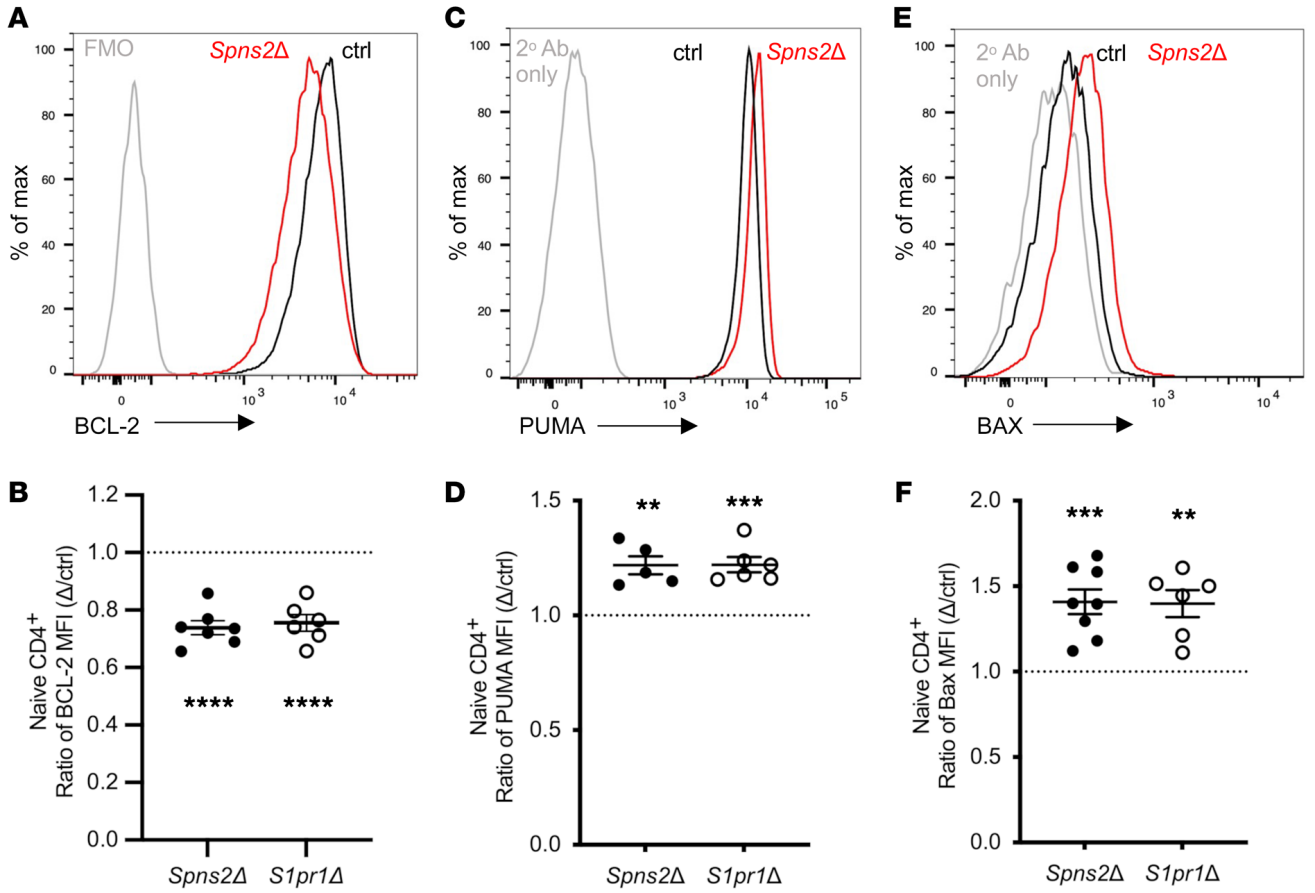
BCL2 family proteins regulate S1PR1-dependent naive T cell survival. Expression of BCL2 family members by naive CD4^+^ T cells from LNs of *Spns2Δ* or *S1pr1Δ* mice and littermate controls, analyzed by flow cytometry. (**A** and **B**) Representative histogram from a *Spns2^fl/fl^* *Lyve1*-Cre mouse and its littermate control, and compilation of BCL2 expression. Each point represents the ratio of BCL2 MFI between a *Spns2Δ* or *S1pr1Δ* mouse and its littermate control (or the mean of littermate controls if there were more than one). Compilation of 5–6 experiments with *n* = 5–7 per group. FMO, fluorescence minus one; cells were stained with all antibodies except anti-BCL2. (**C** and **D**) As in **A** and **B**, for PUMA. Compilation of 5–6 experiments with *n* = 5–6 pairs per group. (**E** and **F**) As in **A** and **B**, for BAX. Compilation of 6 experiments with *n* = 5–8 pairs per group. Student’s *t* test, comparing (KO MFI)/(mean ctrl MFI) vs. (ctrl MFI)/(mean ctrl MFI). **P ≤ 0.01, ***P ≤ 0.001, ****P ≤ 0.0001.

Analysis of RNA and protein indicated that the balance of BCL2 family members was altered upon loss of S1P signaling. Using RNA-Seq, we compared transcripts of *S1pr1Δ^UBC^* and littermate control naive T cells, isolated from mixed bone marrow chimeras in which *S1pr1^fl/fl^ UBC*-CreERT2 and control bone marrow was used to reconstitute WT hosts. We also compared transcripts of naive T cells from chimeras in which WT bone marrow was used to reconstitute either *Spns2Δ^Lyve1^* or littermate control hosts. Two genes within the BCL2 family were altered upon loss of S1P signaling: the anti-apoptotic gene *Bcl2* (BCL2) was downregulated (more consistently in SPNS2-deficient than in S1PR1-deficient T cells), and the pro-apoptotic gene *Bbc3* (PUMA) was upregulated (Supplemental Figure 3A). Using flow cytometry to measure intracellular protein, we found that naive T cells from *Spns2Δ* and *S1pr1Δ* mice had an approximately 20%–25% reduction in BCL2 staining compared with littermate controls (Figure 3, A and B, and Supplemental Figure 3D). Furthermore, we detected an approximately 20%–25% increase in PUMA staining (Figure 3, C and D, and Supplemental Figure 3E). Ultimately, apoptosis is executed by effectors, either BAX or BAK. Naive T cells from *Spns2Δ* and *S1pr1Δ* mice had an approximately 30% increase in BAX staining compared with controls (Figure 3, E and F, and Supplemental Figure 3F). Because T cells are thought to be “primed” for apoptosis, this imbalance of BCL2 family members might trigger cell death via the mitochondrial pathway of apoptosis.

**Figure 4:**
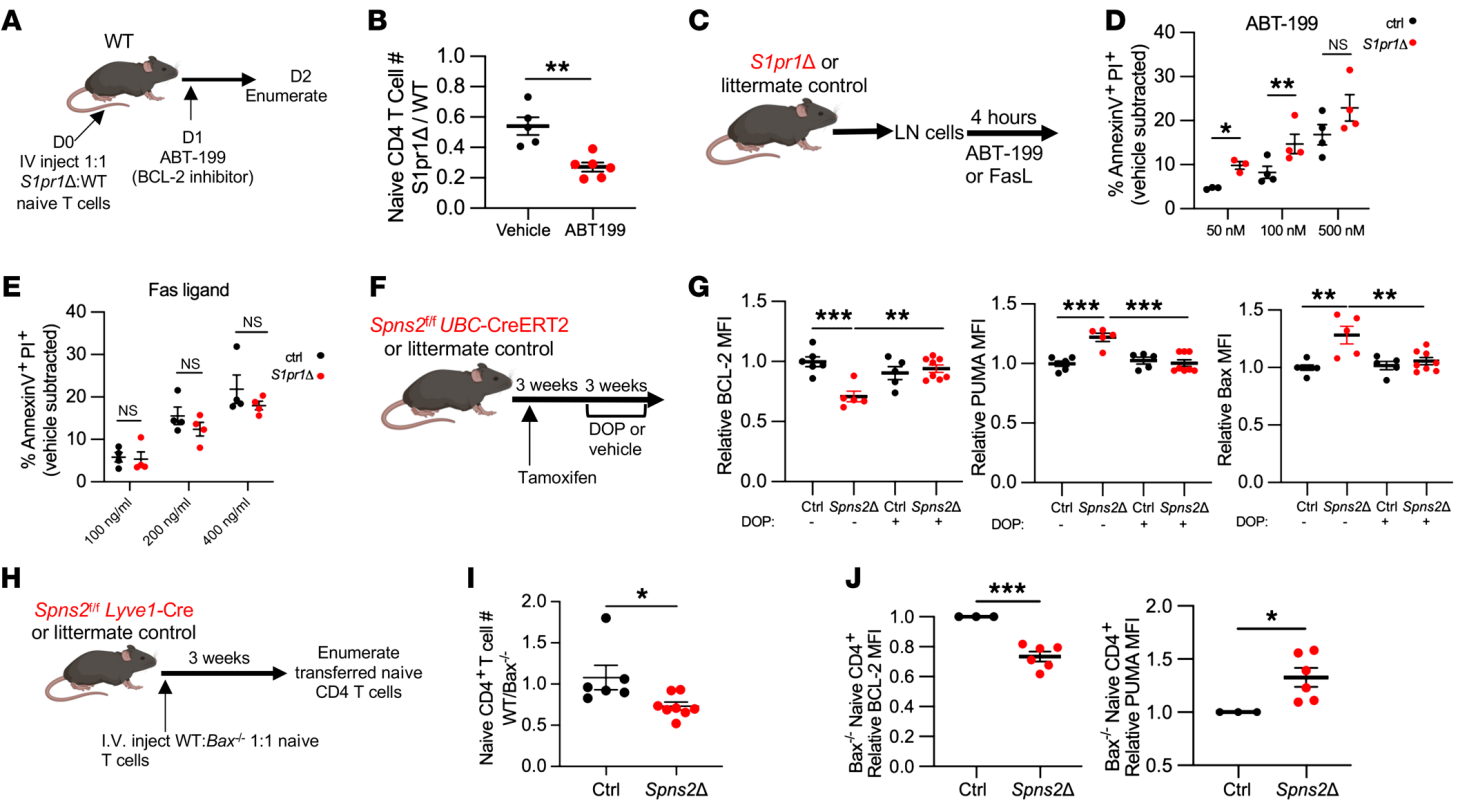
BCL2 family proteins regulate S1PR1-dependent naive T cell survival. (**A** and **B**): (**A**) *S1pr1Δ* and littermate control lymphocytes labeled with CellTraceViolet or CellTraceYellow (dyes swapped between experiments) were cotransferred (1:1 for naive CD4^+^ T cells) i.v. into WT recipients. Twenty-four hours later, recipients were treated with ABT-199; 24 hours later, dye-labeled naive CD4^+^ T cells in LNs were enumerated. (**B**) Ratio of the number of naive CD4^+^ T cells from *S1pr1Δ* donor versus control donor recovered in indicated recipients. Compilation of 3 experiments, 5–6 per group. (**C**–**E**) Lymphocytes from LNs of *S1pr1Δ* or littermate control mice were treated with indicated concentrations of ABT-199 (**D**) or Fas ligand (**E**) ex vivo for 4 hours. Frequency of annexin V^+^ PI^+^ cells among naive CD4^+^ T cells measured by flow cytometry. Each point represents mean of 2 technical replicates, minus mean frequency of annexin V^+^ PI^+^ cells in 2 vehicle-treated technical replicates. Compilation of 3 experiments, 4 per group. (**F** and **G**): (**F**) Experiment design. (**G**) Relative BCL2, PUMA, and BAX expression in naive CD4^+^ T cells, shown as in Figure 3, B, D, and F. Compilation of 4 experiments, *n* = 5–8 per group. (**H** and **I**): (**H**) *Bax^–/–^* and WT littermate control lymphocytes labeled with CellTraceViolet or CellTraceYellow (dyes swapped between experiments) were cotransferred (1:1 for naive CD4^+^ T cells) i.v. into *Spns2Δ* mice and littermate controls; 21 days later, cells in skin-draining and mesenteric LNs were enumerated. (**I**) Ratio of WT/*Bax^–/–^* naive CD4^+^ T cells in indicated mice. Compilation of 3 experiments, 6–8 per group. (**J**) CellTraceViolet-labeled *Bax^–/–^* lymphocytes were transferred i.v. into *Spns2^fl/fl^* *Lyve1*-Cre mice or littermate controls; 21 days later, dye-labeled LN naive CD4^+^ T cells were analyzed by flow cytometry. Compilation of BCL2 and PUMA expression by *Bax^–/–^* naive T cells, as in Figure 3. Compilation of 3 experiments, 3–6 per group. **B**, **D**, **E**, and **I**, Student’s *t* test; **G**, 1-way ANOVA with multiple comparisons. *P ≤ 0.05, **P ≤ 0.01, ***P ≤ 0.001.

To assess whether an imbalance of BCL2 family members resulted in death upon loss of S1P signaling, we tested 3 predictions. First, we predicted that if *S1pr1Δ* T cells had a physiologically relevant imbalance of BCL2 family members, providing an additional stress that activated the mitochondrial pathway of apoptosis would lead to increased death of *S1pr1Δ* T cells compared with controls. We cotransferred *S1pr1Δ* and littermate control naive T cells at a 1:1 ratio into WT recipients. One day after transfer, we treated the recipients with the BCL2-specific inhibitor ABT-199 (29), and 1 day after ABT-199 treatment, we enumerated transferred cells in LNs (Figure 4A). We found that ABT-199 treatment led to a marked reduction in the ratio of *S1pr1Δ* to control T cells (Figure 4B). Similar in vivo experiments using dexamethasone or irradiation also led to a reduction in the ratio of *S1pr1Δ* to control T cells, suggesting that *S1pr1Δ* T cells were more susceptible than controls to pro-apoptotic stresses (Supplemental Figure 3, G and H). ABT-199 treatment ex vivo also led to increased cell death among *S1pr1Δ* cells compared with controls (Figure 4, C and D). By contrast, ex vivo treatment with Fas ligand led to similar cell death among *S1pr1Δ* T cells and controls (Figure 4, C and E), suggesting that *S1pr1Δ* T cells were not more susceptible to death via the extrinsic pathway of apoptosis.

Second, we predicted that if loss of S1P signaling led to an imbalance of BCL2 family members, then increasing T cell exposure to S1P in *Spns2Δ* mice should restore the balance. To test this, we took advantage of the finding that the enzyme S1P lyase maintains low levels of S1P in LNs (18). We treated *Spns2Δ^UBC^* and control mice with the S1P lyase inhibitor 4′-deoxyhydropyridoxine (DOP) for 3 weeks (Supplemental Figure 4A). As expected from previous studies (7), DOP treatment of *Spns2Δ* or control mice increased T cell exposure to S1P within LNs, indicated by internalization of S1PR1 (Supplemental Figure 4B). As expected, in *Spns2Δ^UBC^* mice, increased LN S1P did not restore exit from LNs; in control mice, increased LN S1P disrupted the gradient that would normally guide cells out of LNs and limited exit (Supplemental Figure 4C). As expected, DOP-treated *Spns2Δ^UBC^* mice had a reduced frequency of dying naive T cells compared with vehicle-treated *Spns2Δ^UBC^* mice (Supplemental Figure 4, D–F). We assessed how DOP affected the levels of BCL2 family members in naive T cells. We found that DOP treatment restored BCL2, PUMA, and BAX levels in naive T cells from *Spns2Δ* mice to levels similar to those in controls, consistent with the hypothesis that S1P signaling maintains the balance of BCL2 family members in naive T cells (Figure 4, F and G).

Last, we predicted that the imbalance of BCL2 family members would be the upstream cause of apoptosis in mice lacking S1P signaling. To test this, we took advantage of *Bax^–/–^* T cells (30), which lack one of the two pore-forming proteins that execute the mitochondrial pathway of apoptosis. We cotransferred T cells from *Bax^–/–^* mice and WT littermates into *Spns2Δ^Lyve1^* mice and littermate controls. Three weeks later, we enumerated the transferred cells (Figure 4H). Transferred WT and *Bax^–/–^* T cells were found in similar numbers in WT LNs, while WT T cells decreased about 30% compared with *Bax^–/–^* T cells in *Spns2Δ^Lyve1^* LNs (Figure 4I). Although *Bax^–/–^* T cells survived better in *Spns2Δ^Lyve1^* mice than in control mice, *Bax^–/–^* T cells transferred into *Spns2Δ* mice still had lower levels of BCL2 and higher levels of PUMA than *Bax^–/–^* T cells transferred into controls (Figure 4J and Supplemental Figure 4G). This result suggested that imbalance of these proteins preceded death.

These data suggest that lack of S1P signaling leads to an imbalance in BCL2 family proteins, which promotes naive T cell apoptosis. Of course, additional changes in BCL2 family members beyond those that we measured may also contribute to this imbalance.

### S1P signaling regulates the balance of BCL2 family members by restraining JNK activation.

**Figure 5:**
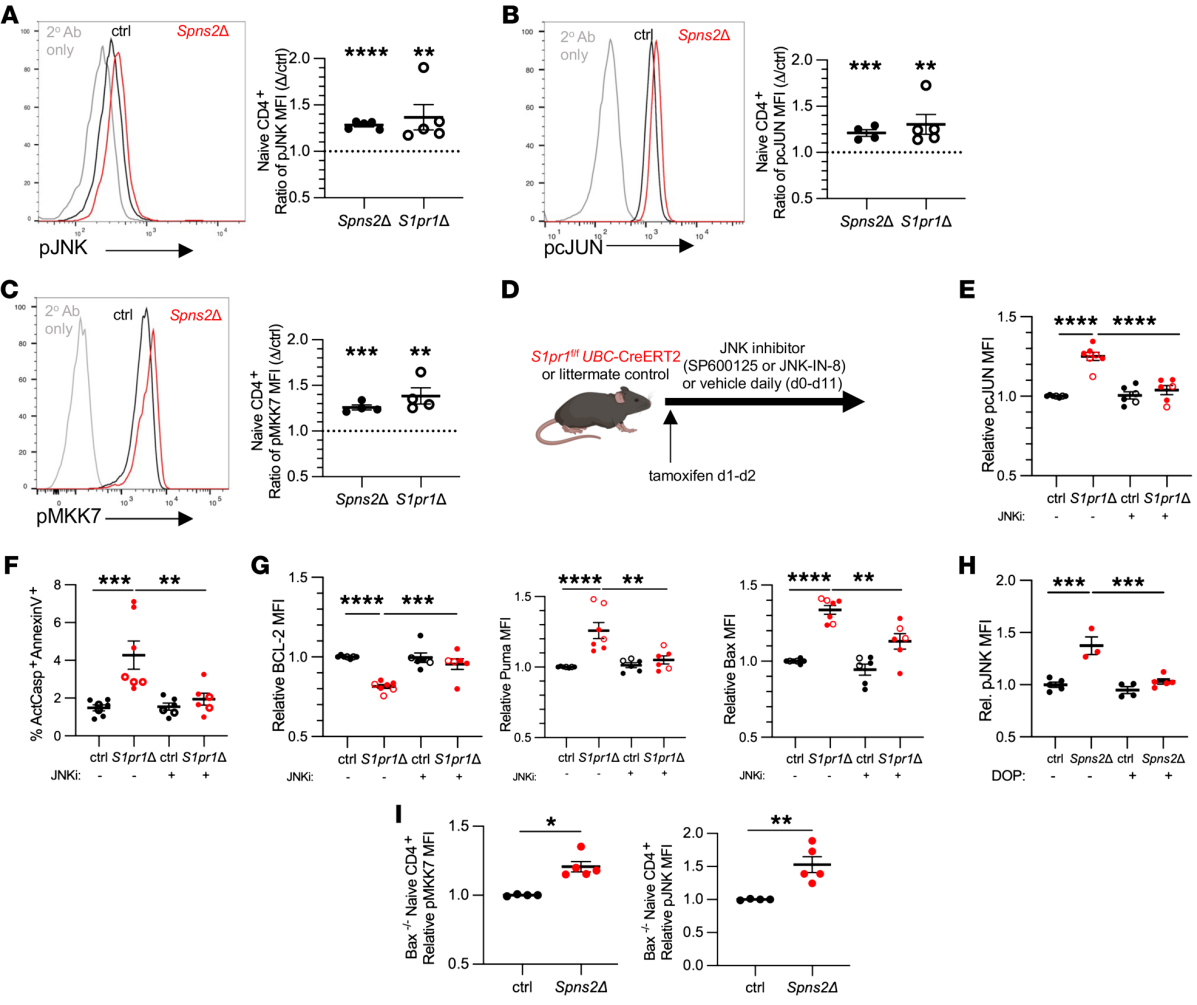
JNK signaling regulates S1P-dependent naive T cell survival. (**A**) Representative histogram from a *Spns2^fl/fl^* *Lyve1*-Cre mouse and littermate control, and compilation of p-JNK expression. Each point represents the ratio of p-JNK MFI between a *Spns2Δ* or *S1pr1Δ* mouse and its littermate control (or mean of littermate controls, if more than one). Compilation of 4–5 experiments, 5–6 per group. (**B**) As in **A**, for p-cJun. Compilation of 4–5 experiments, 5–6 per group. (**C**) As in **A**, for p-MKK7. Compilation of 4 experiments, 4–5 per group. (**D**–**G**) Starting on day 0, *S1pr1^fl/fl^ UBC*-CreERT2 and littermate control mice were treated daily with 15 mg/kg SP600125 (filled circles) or 20 mg/kg JNK-IN-8 (open circles) or vehicle. On days 1 and 2, the mice were treated with tamoxifen. On day 12, naive CD4^+^ T cells in LNs were analyzed. Relative values represent expression in 1 mouse divided by the mean for vehicle-treated controls in that experiment. (**E**) Relative p-cJun expression. (**F**) Frequency of ActCasp^+^ annexin V^+^ among naive CD4^+^ T cells. (**G**) Relative BCL2, PUMA, and BAX expression. Compilation of 6 experiments (4: SP600125; 2: JNK-IN-8), 6–7 per group. (**H**) *Spns2^fl/fl^ UBC*-CreERT2 mice and littermate controls were treated with tamoxifen. Three weeks later, mice were treated with 30 mg/L DOP and 10 mg/L sucrose, or sucrose alone, in drinking water. After 3 weeks of treatment, naive CD4^+^ T cells in LNs were stained for p-JNK. Relative values as in **A**. Compilation of 3 experiments, 3–5 per group. (**I**) CellTraceViolet-labeled *Bax^–/–^* lymphocytes were transferred i.v. into *Spns2^fl/fl^ Lyve1*-Cre mice or littermate controls; 21 days later, dye-labeled naive CD4^+^ T cells in LNs were analyzed by flow cytometry. Compilation of relative p-JNK and p-MKK7 expression by *Bax^–/–^* naive T cells. Relative values represent the MFI in 1 mouse divided by the mean MFI for controls in that experiment. Compilation of 2 experiments, 4–5 per group. **A**–**C** and **I**, Student’s *t* test; **E**–**H**, 1-way ANOVA with multiple comparisons. *P ≤ 0.05, **P ≤ 0.01, ***P ≤ 0.001, ****P ≤ 0.0001.

The expression and function of BCL2 family members are regulated by multiple pathways, including JNK signaling (31). Prolonged JNK activation can promote apoptosis (32). We therefore tested whether JNK was activated in cells lacking S1P signaling. We measured phosphorylation of JNK and one of its downstream targets, the transcription factor cJun, by flow cytometry. Naive T cells from LNs of both *Spns2Δ* and *S1pr1Δ* mice had reproducibly increased expression of phosphorylated JNK1/2 (p-JNK) and phosphorylated cJun (p-cJun) compared with controls (Figure 5, A and B, and Supplemental Figure 5, A and B). JNK can be activated by the kinases MKK4 and MKK7 (32), and we detected increased phosphorylated MKK7 (p-MKK7) in naive T cells from *Spns2Δ* and *S1pr1Δ* mice (Figure 5C and Supplemental Figure 5C).

To assess whether JNK activation resulted in the imbalance of BCL2 family members and increased apoptosis in the absence of S1P signaling, we tested 3 predictions. First, we predicted that decreasing JNK activity would restore levels of BCL2 family members and T cell survival. We treated *S1pr1Δ* mice with one of two structurally distinct JNK inhibitors, SP600125 (33) or JNK-IN-8 (34), or vehicle for 10 days (Figure 5D). JNK inhibition effectively reduced levels of p-cJun (Figure 5E). We found that JNK inhibition decreased the frequency of dying naive T cells in LNs (Figure 5F), and this was accompanied by increased BCL2 and decreased PUMA and BAX (Figure 5G).

Second, we predicted that if loss of S1P signaling led to JNK activation, then increasing T cell exposure to S1P in *Spns2Δ* mice should reduce p-JNK. We treated *Spns2Δ^UBC^* and littermate control mice with DOP for 3 weeks to increase LN S1P. We found that DOP treatment restored p-JNK in naive T cells from *Spns2Δ* mice to levels similar to controls (Figure 5H).

Last, we predicted that JNK activation would be the upstream cause of apoptosis in mice lacking S1P signaling. To test this, we again took advantage of *Bax^–/–^* T cells, which partly recovered in numbers in *Spns2Δ* hosts. We transferred *Bax^–/–^* lymphocytes into *Spns2Δ* mice or controls and measured levels of p-JNK and p-MKK7 three weeks later. *Bax^–/–^* T cells in *Spns2Δ* mice had elevated p-JNK and p-MKK7 compared with *Bax^–/–^* T cells in controls (Figure 5I and Supplemental Figure 5D), suggesting that JNK activation preceded death.

These results suggest that loss of S1P signaling results in JNK activation, which in turn alters the balance of BCL2 family members and leads to apoptosis.

### JNK activation is not explained by loss of mitochondria.

JNK can be activated by many types of cellular stress (32). We had previously observed that *S1pr1Δ* naive T cells had decreased mitochondrial content (7), and we asked whether altered mitochondria could be the key stressor. We analyzed mitochondria in naive T cells by electron microscopy (Supplemental Figure 5E). We found that mitochondria in *S1pr1Δ* naive T cells had decreased cross-sectional area compared with mitochondria in controls, and that *S1pr1Δ* naive T cells had a slightly increased number of mitochondria per cell compared with controls (Supplemental Figure 5, F–I). However, we did not detect differences in cross-sectional area of mitochondria or number of mitochondria in naive T cells from *Spns2Δ* mice compared with controls (Supplemental Figure 5, F–I). We did not observe striking differences in mitochondrial cristae organization between either *S1pr1Δ* mice or *Spns2Δ* mice and controls. While the mitochondrial changes are very interesting, they are unlikely to explain JNK activation, because they are divergent between *S1pr1Δ* and *Spns2Δ* T cells.

### JNK activation correlates with loss of S1PR1 internalization.

To address how S1P and S1PR1 restrain JNK, we turned to our RNA-Seq data sets. Interestingly, we found dysregulation of genes related to endocytosis and the actin cytoskeleton in both T cells lacking S1PR1 and T cells from SPNS2-deficient mice (Supplemental Figure 3, B and C). One striking quality of S1PR1 is that it is extremely susceptible to ligand-induced endocytosis by concentrations of S1P encountered in vivo. During a T cell’s continual trafficking among lymphoid organs, S1PR1 is cyclically lost from the cell surface in the high-S1P environment of blood and lymph, and returned to the cell surface in the low-S1P environment of lymphoid organs (35). We therefore considered the possibility that the constant endocytosis of the receptor itself was important for cell physiology, perhaps by affecting the actin cytoskeleton. Our data so far were consistent with a requirement for S1PR1 internalization in T cell survival. In *Spns2Δ* mice, where naive T cells die, T cells in the LNs maintain very high levels of surface S1PR1 due to lack of S1P exposure. In *Spns2Δ* mice treated with DOP or SEW-2871, naive T cell survival is restored, and surface S1PR1 levels are reduced by ligand-induced internalization.

**Figure 6:**
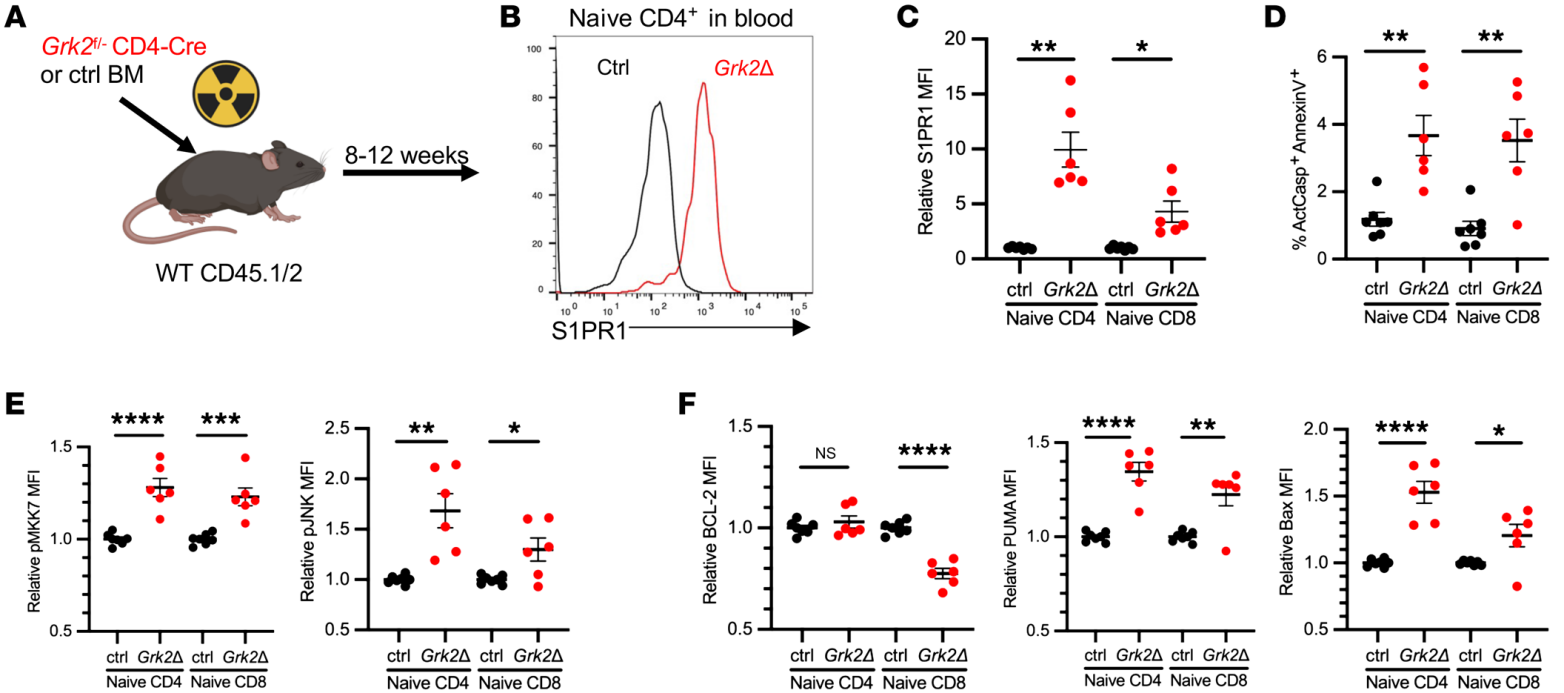
GRK2 regulates naive T cell survival. (**A**) CD45.1/2 WT mice were lethally irradiated and reconstituted with either *Grk2^fl/–^ Cd4*-Cre (*Grk2Δ*) or *Grk2^fl/+^* *Cd4*-Cre (ctrl) bone marrow. Eight to twelve weeks after bone marrow transfer, naive T cells were analyzed. (**B** and **C**) Representative histogram of surface S1PR1 on naive CD4^+^ T cells in blood (**B**), and compilation (**C**). (**D**) Frequency of ActCasp^+^ annexin V^+^ cells among naive CD4^+^ T cells in LNs. (**E** and **F**) Relative MFI values for p-MKK7 and p-JNK (**E**) and for BCL2, PUMA, and BAX (**F**). Relative values represent expression in 1 mouse divided by the mean for littermate controls in that experiment. Compilation of 4 experiments, 6–7 per group. Student’s *t* test. *P ≤ 0.05, **P ≤ 0.01, ***P ≤ 0.001, ****P ≤ 0.0001.

To test whether internalization of S1PR1 was important for naive T cell survival, we took advantage of the knowledge that the kinase GRK2 induces S1PR1 internalization by phosphorylating the receptor’s C-terminal tail and enabling β-arrestin to bind the receptor (36, 37). In *Grk2^fl/–^* CD4-Cre mice, naive T cells maintain aberrantly high levels of S1PR1 in blood and are present at reduced numbers in blood and secondary lymphoid organs (37). We generated bone marrow chimeras by reconstituting WT mice with congenically marked *Grk2^fl/–^* CD4-Cre (*Grk2Δ*) or *Grk2^fl/+^* CD4-Cre (control) bone marrow (Figure 6A). Eight weeks after reconstitution, we confirmed increased S1PR1 levels on naive CD4^+^ T cells in blood (Figure 6, B and C). We found that LNs of *Grk2Δ* mice had an increased frequency of dying naive T cells, accompanied by increased JNK activation and an imbalance of BCL2 family members, with the exception of similar BCL2 expression between *Grk2Δ* and control naive CD4^+^ T cells (Figure 6, D–F). These results are largely consistent with the possibility that deficient S1PR1 internalization impairs naive T cell survival, but interpretation is complicated by pleiotropic functions of GRK2.

**Figure 7:**
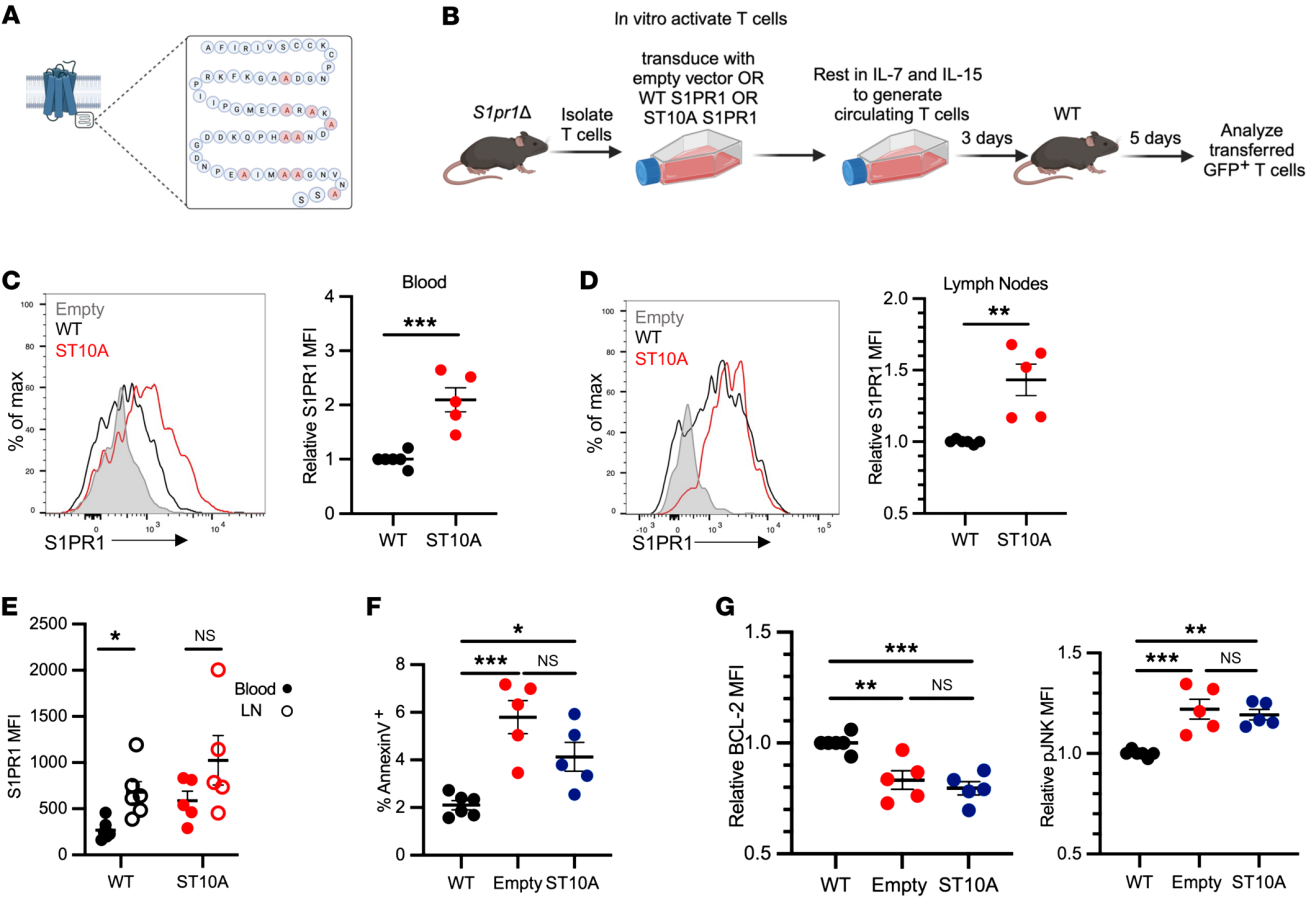
Loss of S1PR1 internalization contributes to apoptosis. T cells were isolated from LNs of *S1pr1Δ* mice, activated in vitro with anti-CD3/CD28, and retrovirally transduced with vector encoding either IRES-GFP (empty vector), WT S1PR1-IRES-GFP, or ST10A S1PR1-IRES-GFP. Transduced T cells were cultured for 3 days in IL-7/IL-15 medium to generate predominantly CD8^+^ “central memory–like” T cells. These T cells were transferred into WT recipients, and 5 days later, transferred CD8^+^ T cells were analyzed. (**A**) Schematic of ST10A. (**B**) Experiment design. (**C** and **D**) Representative histogram and compilation of relative surface S1PR1 expression on cells in blood and LNs. Relative surface S1PR1 represents MFI on cells in 1 mouse divided by the mean MFI on WT S1PR1-transduced T cells in that experiment. (**E**) Absolute S1PR1 MFI values on the indicated cells. (**F**) Frequency of annexin V^+^ cells among CD8^+^ GFP^+^ T cells in LNs. (**G**) Relative MFI for BCL2 and p-JNK in CD8^+^ GFP^+^ T cells in LNs. Relative values represent expression in 1 mouse divided by the mean for WT S1PR1-transduced cells in that experiment. Compilation of 5 experiments, 5–6 per group. **C**–**E**, Student’s *t* test; **F** and **G**, 1-way ANOVA with multiple comparisons. *P ≤ 0.05, **P ≤ 0.01, ***P ≤ 0.001.

To study S1PR1 internalization more specifically, we manipulated the C-terminal tail of S1PR1, which has a series of serine and threonine residues that are phosphorylated by GRK2. Replacement of 5 serines in the C-terminal tail with alanine (S351A, S353A, S355A, S358A, S359A) (38) or of 2 serines and 1 threonine in the C-terminal tail with alanine (T371A, S374A, S375A) (37) had been shown to slow S1PR1 internalization, but in both cases surface S1PR1 remained undetectable on T cells in blood and no abnormalities in T cell numbers were reported. To more completely limit internalization, we mutated S1PR1 to substitute alanine for 9 serines and 1 threonine in the C-terminal tail (ST10A) (Figure 7A). Naive *S1pr1Δ* T cells were activated in culture; transduced with empty vector, S1PR1-WT, or S1PR1-ST10A; then rested for 3 days in IL-7 and IL-15 to generate CD8^+^ T cells with central memory properties (39). Empty vector–, S1PR1-WT–, or S1PR1-ST10A–expressing *S1pr1Δ* T cells were transferred into recipients. Four to five days later, the transferred T cells were analyzed (Figure 7B). S1PR1-ST10A–transduced *S1pr1Δ* T cells had higher levels of surface S1PR1 compared with S1PR1-WT–transduced *S1pr1Δ* T cells in blood and LNs (Figure 7, C–E). While S1PR1-WT–transduced *S1pr1Δ* T cells had lower rates of apoptosis than empty vector–transduced *S1pr1Δ* T cells, S1PR1-ST10A–transduced T cells had similarly high rates of apoptosis to empty vector–transduced *S1pr1Δ* T cells (Figure 7F). Similarly, while S1PR1-WT–transduced *S1pr1Δ* T cells had lower levels of p-JNK and higher levels of BCL2 than empty vector–transduced *S1pr1Δ* T cells, S1PR1-ST10A–transduced T cells had similar levels of p-JNK and BCL2 to empty vector–transduced *S1pr1Δ* T cells (Figure 7G).

Taken together, these data suggest that phosphorylation of the C-terminal tail of S1PR1 regulates both receptor internalization and T cell survival, consistent with the possibility that endocytosis of S1PR1 restrains JNK activation, maintains the balance of BCL2 family members, and prevents apoptosis.

### Chronic FTY720 treatment recapitulates apoptosis.

S1PR1 modulators have had remarkable clinical efficacy treating multiple sclerosis and ulcerative colitis (UC). FTY720, the most commonly used drug targeting S1PR1, acts as an agonist of S1PR1 in the short term but as a functional antagonist of S1PR1 in the long term in many contexts. After binding S1PR1 and inducing an acute signal through the receptor, FTY720 induces S1PR1 internalization and degradation, mimicking genetic loss of *S1pr1* (40). By contrast, S1P or SEW-2871 induces S1PR1 signaling and internalization, but S1PR1 is shuttled back to the cell surface, where it can signal again (40). Recent studies measuring blood lymphocyte counts in patients who have stopped FTY720 treatment suggest that some patients do not fully recover cell numbers months after treatment cessation (13, 14). We hypothesized that FTY720 may not only block lymphocyte circulation but also induce naive T cell death.

**Figure 8:**
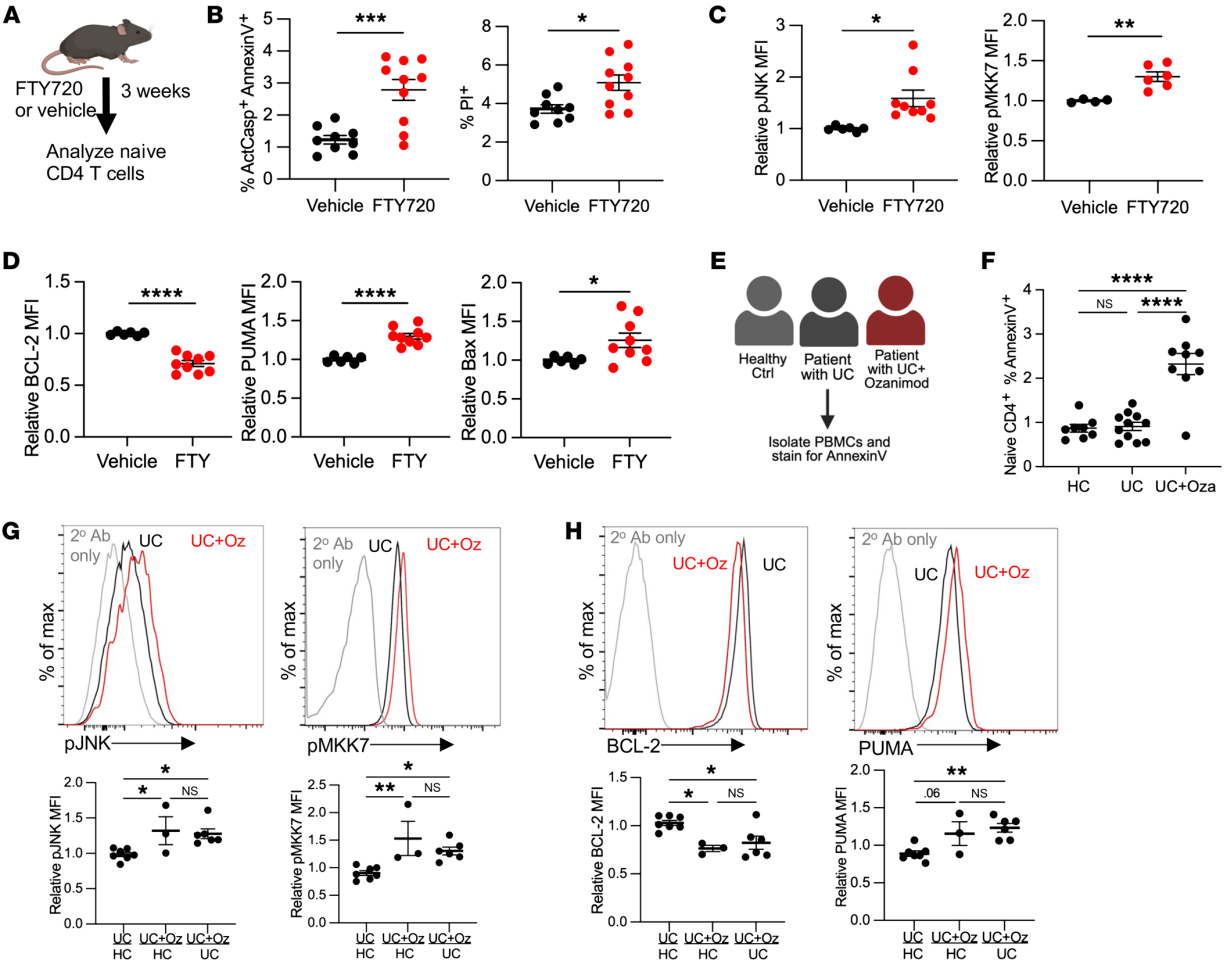
S1PR1 modulators impair T cell survival in mice and humans. (**A**–**D**): (**A**) WT mice were treated with 1 mg/kg FTY720 or vehicle every other day for 3 weeks, and naive CD4^+^ T cells in LNs were analyzed by flow cytometry. (**B**) Frequency ActCasp^+^ annexin V^+^ and PI^+^ among naive CD4^+^ T cells in LNs. Compilation of 3 experiments, 9–10 per group. (**C**) Relative MFI for p-MKK7 and p-JNK. Relative MFI represents MFI in 1 mouse divided by the mean for controls in that experiment. p-MKK7: compilation of 2 experiments, 4–6 per group; p-JNK: compilation of 3 experiments, 6–9 per group. (**D**) Relative MFI for BCL2, PUMA, and BAX. Compilation of 3 experiments, 6–9 per group. (**E**–**H**): (**E**) PBMCs were isolated from UC patients treated with ozanimod (UC+Oza), UC patients not treated with ozanimod (UC), or healthy controls (HC), and analyzed by flow cytometry. (**F**) Frequency annexin V^+^ among naive CD4^+^ T cells. Compilation of 10 experiments, 8–11 per group. (**G**) Representative histograms and compilations of p-JNK and p-MKK7 relative MFI for naive CD4^+^ T cells. Relative MFI represents MFI in 1 sample divided by the mean for the indicated comparison group on that day. Left column: UC patients not treated with ozanimod versus healthy controls. Middle column: UC patients treated with ozanimod versus healthy controls. Right column: UC patients treated with ozanimod versus UC patients not treated with ozanimod. Each UC patient with ozanimod is represented once; if there was a choice between comparing that patient with a healthy control and comparing him or her with a UC patient not taking ozanimod, he or she was compared with the UC patient not taking ozanimod. Compilation of 10 experiments, 3–7 per group. (**H**) Representative histograms and compilation of relative MFI for BCL2 and PUMA. Compilation of 10 experiments, 3–7 per group. **B**–**D**, Student’s *t* test; **F**–**H**, 1-way ANOVA with multiple comparisons. *P ≤ 0.05, **P ≤ 0.01, ***P ≤ 0.001, ****P ≤ 0.0001.

To test this, we treated WT mice with FTY720 or vehicle for 3 weeks (Figure 8A). We found an increased frequency of dying cells among naive CD4^+^ T cells in LNs of FTY720-treated mice compared with controls, accompanied by increased p-JNK and p-MKK7, decreased BCL2, and increased PUMA and BAX (Figure 8, B–D). Although FTY720 has pleiotropic effects, including blockade of thymic exit, these data are consistent with FTY720 acting as a functional antagonist of S1PR1 in the context of T cell death.

We next asked whether increased T cell death was also present in patients treated with S1PR1 modulators. We analyzed peripheral blood mononuclear cells (PBMCs) from UC patients being treated with the S1PR1 modulator ozanimod (9) (Figure 8E, Supplemental Table 1, and Supplemental Figure 6A). We found an increased percentage of annexin V^+^ cells among naive CD4^+^ T cells as well as naive CD8^+^ and central memory CD4^+^ T cells in patients treated with ozanimod compared with healthy controls or UC patients on a different therapy (Figure 8F and Supplemental Figure 6B). We also detected increased activation of the JNK pathway and an imbalance of BCL2 family members in T cells from ozanimod-treated patients compared with controls (Figure 8, G and H, and Supplemental Figure 6, C and D). These data suggest that loss of S1PR1 function in human T cells, as in murine T cells, not only disrupts their trafficking but also may lead to apoptosis.

### Chronic FTY720 treatment reduces T follicular helper and germinal center B cell numbers.

In response to the SARS-CoV-2 vaccine, patients on S1PR1 modulators have lower levels of anti-spike IgG antibody and lower seroconversion rates compared with patients treated with most other types of immune suppressants (41). Interestingly, the strength of the antibody response has been reported to correlate inversely with the duration of drug treatment before vaccination (11, 12), while effects of these drugs on lymphocyte trafficking should be rapid and sustained. We hypothesized that one mechanism for the reduced response is loss of the naive T cell repertoire due to apoptosis (42).

**Figure 9:**
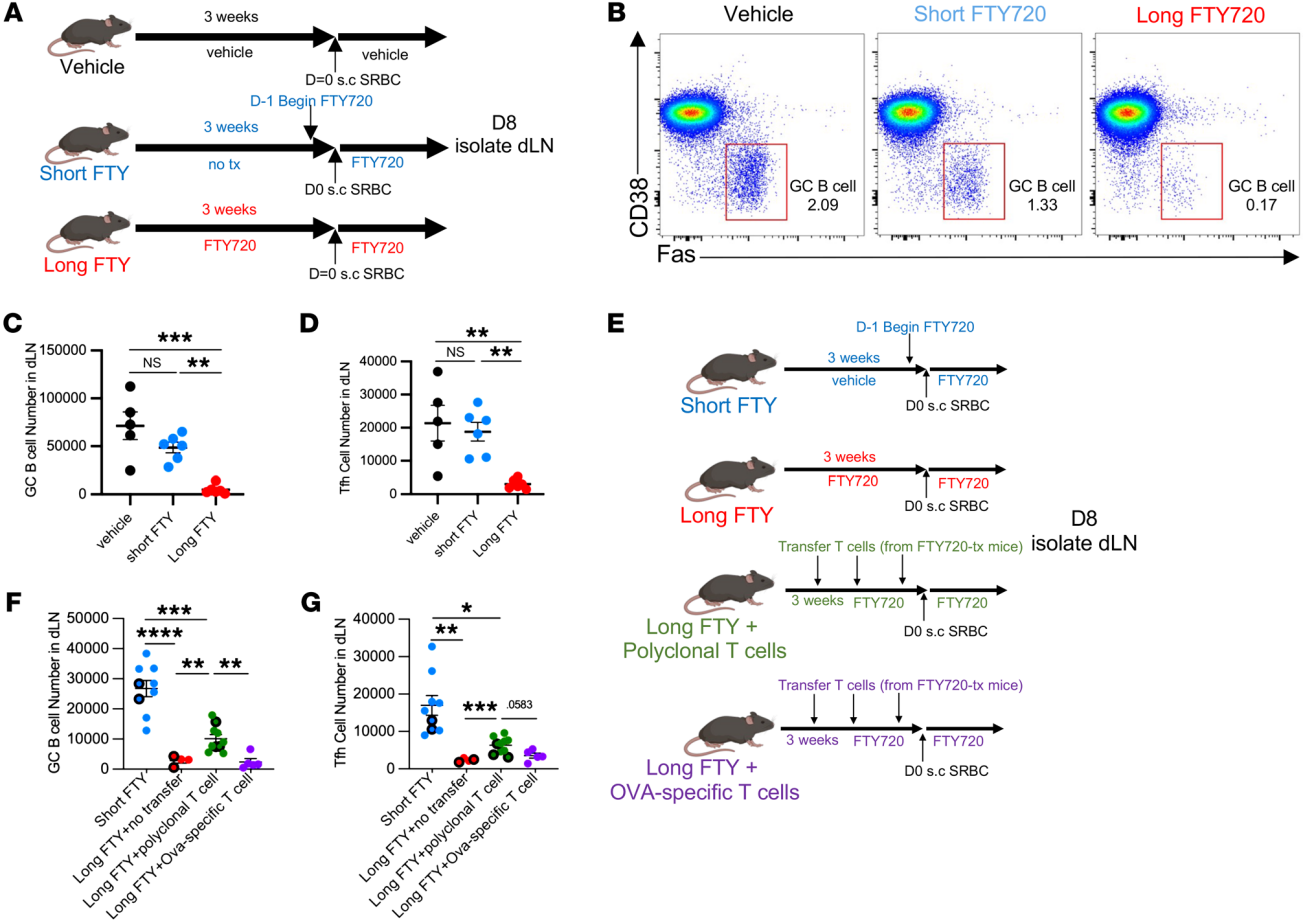
Prolonged FTY720 treatment impairs germinal center responses. (**A**–**D**): (**A**) WT mice were divided into 3 groups: “Long FTY,” 1 mg/kg FTY720 every other day for 3 weeks (starting day –21); “Vehicle,” vehicle for 3 weeks (starting day –21); and “Short FTY,” untreated until day –1, when they received 1 mg/kg FTY720. On day 0, all mice received 10^8^ sheep red blood cells (SRBCs) subcutaneously. FTY720 (long and short groups) or vehicle (vehicle group) treatment continued until day 8, when draining LNs were analyzed. (**B**) Representative plots of GCB cells (Fas^+^CD38^–^), gated on B220^+^ B cells. Numbers represent percentage GC of total B cells. (**C** and **D**) Number of GCB (**C**) or Tfh (**D**) cells in draining LNs. Compilation of 3 experiments, 5–6 per group. (**E**–**G**): (**E**) Three groups of WT mice were treated with FTY720 for 3 weeks. One group (Long FTY + polyclonal T cells) received CD4^+^ and CD8^+^ naive T cells from FTY720-treated WT mice (donor mice initiated FTY720 at the same time as recipient groups), either once (day –1: black-outlined circles) or 3 times (days –15, –8, and –1: filled circles). The second group received OVA-specific CD4^+^ and CD8^+^ T cells from FTY720-treated OT-I and OT-II transgenic mice (donor mice initiated FTY720 at the same time as recipient groups) 3 times (days –15, –8, and –1: filled circles); the OT-I/OT-II ratio matched the CD8^+^/CD4^+^ T cell ratio in the polyclonal transfer. One group received FTY720 at day –1 (Short FTY). On day 0, all mice received 10^8^ SRBCs subcutaneously. FTY720 treatment continued in all groups until day 8, when draining LNs were analyzed. (**F** and **G**) Number of GCB cells (**F**) and Tfh cells (**G**) in draining LNs. Four experiments, 5–10 per group. **C** and **D**, 1-way ANOVA with multiple comparisons; **F** and **G**, Brown-Forsythe and Welch’s ANOVA test. *P ≤ 0.05, **P ≤ 0.01, ***P ≤ 0.001, ****P ≤ 0.0001.

To test this hypothesis, we studied the response of FTY720-treated mice to a T cell–dependent model antigen, sheep red blood cells, injected subcutaneously. We enumerated T follicular helper (Tfh) cells and germinal center B (GCB) cells in the draining LNs 8 days after immunization. We compared vehicle, “short” FTY720 treatment (first dose 1 day before immunization), and “long” FTY720 treatment (first dose 3 weeks before immunization) (Figure 9A). Interestingly, long FTY720 treatment led to a substantial reduction in the numbers of Tfh and GCB cells compared with other groups (Figure 9, B–D, and Supplemental Figure 6E).

FTY720 targets multiple S1P receptors on numerous cell types (43, 44), any of which might contribute to the declining germinal center response over time. To test whether the reduced germinal center response was due to loss of the naive T cell repertoire (which in FTY720-treated mice, as in patients, was due to both cell death and a loss of thymic exit), we asked whether we could restore the response by increasing the number of naive T cells (Figure 9E). We transferred polyclonal naive T cells into FTY720-treated mice. As a control, we transferred the same number of ovalbumin-specific T cells into a second group of FTY720-treated mice. If the decreased germinal center response were due to a loss of the naive T cell repertoire, ovalbumin-specific T cells should not restore it. The transferred cells were isolated from donors treated with FTY720 using the same protocol as recipients, to avoid unanticipated artifacts. Transfer of polyclonal T cells, but not ovalbumin-specific T cells, increased Tfh and GCB cell numbers (Figure 9, F and G). The rescue was incomplete, but we were unable to replenish the T cell pool fully. The number of GCB cells increased with the total number of T cells in the case of polyclonal T cell transfer (Supplemental Figure 6F). As expected, transfer of ovalbumin-specific T cells did not result in a large increase in the total number of T cells, likely owing to competition for a limited niche (45) (Supplemental Figure 6F).

## Discussion

Naive T cell survival is crucial to maintain a diverse repertoire of T cells, which enables a robust immune response to a diverse range of pathogens. Here, we addressed how S1P and S1PR1 support naive T cell survival, and whether this pathway is disrupted in patients treated with S1PR1 antagonists. We found that S1P signaling through S1PR1 limits JNK activation, which in turn maintains the appropriate balance of BCL2 family members within the T cell, and thereby prevents apoptosis. Interestingly, T cells without S1P/S1PR1 signaling have differential expression of genes regulating endocytosis and the actin cytoskeleton compared with controls, and phosphorylation of the same residues of the S1PR1 C-terminus that enable ligand-induced internalization of S1PR1 also promotes S1PR1-dependent survival. FTY720, a functional antagonist of S1PR1 that is used extensively to treat patients with autoimmune disease, induces naive T cell death via the same pathway as genetic deletion of SPNS2 and S1PR1. The loss of naive T cells can limit generation of B cell responses.

Our work suggests that S1PR1 modulators currently used clinically to treat autoinflammatory diseases may induce naive T cell apoptosis. Early studies aimed at understanding the lymphopenia induced by FTY720 found increased apoptosis of cells treated with the drug in vitro (46–48). Notably, one study found increased JNK activation (49), and another study found that BCL2 overexpression prevented FTY720-induced apoptosis (47, 48). Together with many studies implicating S1P in cell survival, these experiments suggested that cell death could account for the lymphopenia and immunosuppression (19, 50, 51). In contrast, in vivo studies demonstrated that FTY720 induced lymphopenia by inhibiting egress of lymphocytes into lymph (44). Our experiments echo the early findings. FTY720’s effects on cell death in vivo may have been discounted as a result of its more readily observable effects on cell trafficking.

One key future direction is to explore how these findings should alter clinical practice. Our work suggests that it will be important to consider effects of S1PR1 modulators on immune responses within the LNs, as well as the more obvious effects on immune responses in non-lymphoid tissues. Reductions in naive T cell numbers may limit the initiation of immune responses, and it will be important in future work to assess how chronic JNK signaling may affect T cell function. In some settings, this may be an unacceptable side effect. In other settings, this may be beneficial, limiting the recruitment of new T cells into an immune response. Going forward, effects of targeting S1PR1 on the immune system must be assessed more holistically.

On the more basic side, an interesting question is how S1PR1 limits JNK activation. Our data suggest that S1PR1 internalization (or the amino acids that enable internalization) restrains JNK. We are considering 3 candidate mechanisms. First, S1PR1 might signal through a unique partner within endosomes or another intracellular compartment, which in turn might limit JNK phosphorylation. Intracellular functions of GPCRs are increasingly appreciated (52); for example, after ligand-induced internalization, GPR35 traffics to the outer mitochondrial membrane, where it regulates protein-protein interactions (53). Second, when S1PR1 cannot be internalized, the internalization machinery might be freed to associate with other receptors, which may result in JNK activation. For example, β-arrestin, a key regulator of GPCR internalization, has a MAP kinase docking site that can enable JNK activation in some settings (54, 55).

Third, loss of S1PR1 internalization might itself induce cell stress, which in turn could result indirectly or directly in JNK activation. Receptor internalization requires rearrangement of the actin cytoskeleton, and stabilization of actin filaments or alterations in actin binding proteins can induce JNK activation (56). Furthermore, S1PR1 has recently been shown to promote a unique form of bleb-based movement by utilizing ezrin-radixin-moesin proteins, which form linkages between the plasma membrane and the actin network (26). Ezrin phosphorylation is associated with activation of MKK7 and JNK in B cells (57), suggesting the possibility that ezrin phosphorylation-dephosphorylation events may regulate actin dynamics and thereby affect cell survival. Interestingly, mice deficient in the actin regulators *Dock8* (58) or *Coro1a* (59, 60) have perturbed T cell homeostasis due to both migration defects and increased cell death. Lymphocytes must be able to deform in order to squeeze through endothelial barriers as well as to move within organs, and perhaps the same receptors that guide their migration keep the cytoskeleton flexible.

While the role of S1PR1 in T cell egress from lymphoid organs is increasingly well understood, to the point at which 5 drugs targeting S1PR1 have been approved by the FDA for treatment of inflammatory disease and more are in clinical trials, the role of S1PR1 in T cell survival has just begun to be explored. It will be essential to understand this pathway to better direct use of this powerful class of drugs.

## Methods

### Study design

For mice, sample sizes balanced statistical robustness and animal welfare, and negative results should not be overinterpreted. No animals were excluded from analysis unless they were clearly sick (hunched, low body weight). No specific method of randomization was used to allocate mice into groups, although sex-matched littermates were used when possible. For human samples, we analyzed blood from all consented patients who were treated with ozanimod at NYU Langone’s Inflammatory Bowel Disease Center (New York, New York, USA) over 16 months, and consented controls who visited the clinic the same day. The order of sample collection and data acquisition was designed to avoid experimental bias: collection and processing of samples from control and knockout, as well as treated and untreated animals and people, were alternated. Investigators were not blinded, because there were no qualitative measurements. No outliers were excluded, and no experiment was excluded unless a positive or negative control failed.

### Mice

C57BL/6J (WT; CD45.2), B6.SJL-*Ptprc^a^Pepc^b^*/BoyJ (CD45.1), *Spns2^fl/fl^* (61), *S1pr1^fl/fl^* (62), *Bax^–/–^* (30), *Lyve1*-Cre (16), *UBC*-CreERT2 (15), MHCII*^–/–^* (63), *Cd69^–/–^* (64), *Sphk1^fl/fl^* (65), *Sphk2^−/−^* (66), OT-I (67), and OT-II (68) mice have been previously described. All mice were bred in NYU Grossman School of Medicine animal facilities. Mice were on a C57BL/6 or, in some cases, a C57BL/6×129 background. Mice were always compared with littermate controls. Mice were 7–20 weeks old at the time of analysis. Male and female mice were used depending on availability, as sex did not seem to affect the results. Mice were housed in specific pathogen–free conditions. All cages were on a 12-hour light/12-hour dark cycle (lights on at 7 am). Room temperature was maintained at 72°F ± 2°F (22.2°C ± 1.1°C), and room humidity was maintained at 30%–70%.

### Human blood samples

Peripheral blood was drawn from anonymous, adult inflammatory bowel disease (IBD) patients being treated with Zeposia (ozanimod), adult IBD patients being treated with a different therapy, or healthy control adults.

### Treatments

#### Tamoxifen.

Tamoxifen (Sigma-Aldrich) was dissolved in corn oil (Sigma-Aldrich) by shaking at 37°C for 3 hours, at 20 mg/mL or 40 mg/mL. Two milligrams tamoxifen was administered intraperitoneally on 5 consecutive days, or 6 mg tamoxifen was administered by gavage on 2 consecutive days. Unless otherwise indicated, analysis was 3–4 weeks after the last tamoxifen treatment. Tamoxifen efficacy was checked by enumeration of blood naive T cells in the case of *Spns2^fl/fl^ UBC*-CreERT2, where we saw naive T cells in blood decline approximately 20-fold. In the case of *S1pr1^fl/fl^ UBC*-CreERT2, tamoxifen efficacy was checked by measurement of surface S1PR1 or surface CD69 by flow cytometry, and deletion efficiency was 80%–95%. We excluded experiments in which deletion was less than 75%. We did not exclude non-deleted cells from analysis except when we sorted cells; this reduced the magnitude of differences, but avoided assumptions.

#### Bone marrow chimera generation.

Recipients were lethally irradiated with two 5 Gy doses of x-ray radiation separated by 3 hours, and received 2 × 10^6^ bone marrow cells intravenously. Chimeras were analyzed at least 8 weeks after transplantation.

#### IL-7Rα blockade.

Mice received 0.4 mg anti–IL-7Rα (Bio X Cell) or isotype control (rat IgG2a) intraperitoneally every 4 days for 3 total treatments.

#### Transfers of Bax^–/–^ or littermate control T cells.

T cells were isolated by negative selection using biotinylated antibodies against CD11b, CD11c, CD19, CD25, NK1.1, TCRγΔ, and Ter119 and Magnisort Negative selection beads (Thermo Fisher Scientific) according to the manufacturer’s instructions, then labeled with CellTraceViolet or CellTraceYellow (Thermo Fisher Scientific). Dyes were swapped between genotypes between experiments. 1 × 10^6^ to 2 × 10^6^ T cells per group were transferred via retro-orbital injection.

#### 4-Deoxypyridoxine-HCl.

Mice received 10 g/L sucrose plus 30 mg/L 4-deoxypyridoxine-HCl (DOP; Sigma-Aldrich) or 10 g/L sucrose alone in drinking water for 3 weeks.

#### SEW-2871.

SEW-2871 (Cayman Chemical) was dissolved in ethanol at 9 mg/mL as stock solution. Stock was diluted in vehicle of 50% Tween-20 to 50% PBS. Solution was sonicated in a water bath for 10 minutes, then injected intraperitoneally at 10 mg/kg daily.

#### H-89.

H-89 (40 mg/kg; Selleckchem) was given by gavage in vehicle of 5% DMSO, 30% PEG400, 1% Tween-80, 64% PBS daily for 12 days.

#### Dexamethasone.

Dexamethasone (Sigma-Aldrich) was injected intraperitoneally in vehicle of PBS at 2 mg/kg.

#### Irradiation.

Adult (8–12 weeks) C57BL/6J mice were exposed to 1 Gy x-ray radiation.

#### ABT-199.

ABT-199 (60 mg/kg; Selleckchem) was given by gavage in vehicle of 8% DMSO, 45% PEG400, 5% Tween-80, 42% PBS.

#### JNK inhibitors.

SP600125 (15 mg/kg/d; Selleckchem) or JNK-IN-8 (20 mg/kg/d; MedChemExpress) was given intraperitoneally in vehicle of 4.5% DMSO, 40% PEG400, 5% Tween-80, and 50.5% PBS daily for 12 days.

#### FTY720.

Mice were injected intraperitoneally with 1 mg/kg FTY720 (Cayman Chemical) in vehicle of 0.5% DMSO, 4.5% 2-hydroxypropyl-b-cyclodextrin, and 95% PBS every other day for 3 weeks or as indicated.

#### Sheep red blood cells.

Sheep red blood cells (SRBCs) (Colorado Serum Company) were washed 3 times in PBS. Washed SRBCs were enumerated using a Beckman Coulter Multisizer 3 and resuspended at 10^8^ in 30 μL. 10^8^ SRBCs were subcutaneously injected into the left footpad, which drains to the popliteal and inguinal LNs.

### Cell preparation

For analysis by flow cytometry, lymphocytes were isolated from spleen or LNs by mechanical disruption and filtration through a 70 μm cell strainer in isolation buffer (PBS with 2% FBS, 1 mM EDTA). LNs were combined cervical, brachial, axillary, inguinal, and mesenteric, unless otherwise indicated. Lymphocytes were enumerated with a cell counter (Beckman Coulter Multisizer 3) set to detect nuclei between 3.5 and 10 μm. For adoptive transfer, T cells were purified by magnetic bead enrichment (Thermo Fisher Scientific) and retro-orbitally injected.

For CellTrace dye labeling, cells were resuspended at 20 × 10^6^ per milliliter in PBS, and dyes were added to a final concentration of 5 μM. Cells were incubated for 10 minutes at room temperature, and the reaction was stopped by addition of PBS-FBS to a final concentration of 20% FBS. Labeled cells were washed twice in PBS before injection into mice.

To generate bone marrow chimeras, bone marrow cells were flushed from long bones of the leg. RBCs were lysed using ACK lysis buffer (Gibco). Cells were resuspended at a concentration of 2 × 10^6^ per 100 μL PBS.

For human cells, PBMCs were isolated using Lymphoprep (StemCell Technologies) and Sepmate-50 (IVD) tubes (StemCell Technologies), according to the manufacturer’s instructions.

### In vitro culture with ABT-199 and Fas ligand

Lymphocytes were treated with either ABT-199 (Tocris) or Fas ligand plus enhancer (Enzo Life Sciences) for 4 hours in a 37°C 5% CO_2_ incubator. After 4 hours, cells were stained for surface markers and then stained for annexin V (BioLegend) and propidium iodide in Annexin V Binding Buffer (BioLegend) for 15 minutes at room temperature.

### Flow cytometry

Staining for S1PR1 was done on ice in PBS with 0.05% sodium azide, 1 mM EDTA, and 0.5% FBS. Cells (2E6 in 25 μL) were stained for 90 minutes with anti–mouse S1PR1 (74.4 μg/mL; R&D Systems), washed twice in buffer, stained for 45 minutes with anti-rat IgG-biotin F(ab′)_2_ (9.5 μg/mL; Jackson ImmunoResearch), washed twice in buffer, and stained with streptavidin-APC and other antibodies (Supplemental Table 2).

To stain for active caspase and annexin V, single-cell suspensions (2E6 cells in 25 μL) were stained for surface markers with 10 μM CaspACE FITC-VAD-FMK for 1 hour in PBS supplemented with 1 mM EDTA and 0.5% FBS on ice. Cells were washed once in previous buffer and once in Annexin V Binding Buffer (BioLegend). Then cells were stained with 1:50 dilution Annexin V (BioLegend) Pacific Blue or APC in Annexin V Binding Buffer for 15 minutes at room temperature.

For intracellular staining, cells were first stained with Fixable Live/Dead Blue (Thermo Fisher Scientific) according to the manufacturer’s instructions. Then, cells were stained for surface markers on ice for 30 minutes. Cells were fixed using eBioscience FoxP3 Fixation/Permeabilization kit either for 1 hour or overnight at 4°C, then stained for intracellular markers for 1 hour. If secondary antibodies were required, this step was performed for 30 minutes.

For phospho-staining, tissues were mechanically disrupted in PBS to create a single-cell suspension and immediately fixed in equal volume of 4% paraformaldehyde for 15 minutes. Cells were washed in PBS with 2% FBS and 1 mM EDTA, then stained for surface markers (CD4 BUV395 [RM4-5], CD8 PE [5.3-6.7], CD62L BV421 [MEL-14], CD44 FITC [IM7], and CD25 BV650 [PC61]). After surface staining, cells were permeabilized using eBioscience FoxP3 permeabilization kit for 1 hour. Cells were stained for phospho-antigens for 1 hour with primary antibodies and then stained using secondary antibodies for 30 minutes.

Flow cytometry data were acquired on an LSR-II flow cytometer (Becton Dickinson) and analyzed using FlowJo v10 software (BD Biosciences).

### RNA isolation and sequencing

RNA was isolated from FACS-sorted naive CD4^+^ T cells using TRIzol LS (Invitrogen) followed by DNase I (QIAGEN) treatment and cleanup with an RNeasy Plus Micro kit (QIAGEN), and quantified on a 2100 BioAnalyzer instrument (Agilent Technologies Inc.). RNA-Seq library preparation and sequencing were performed at the NYU Langone Genome Technology Center. Five to twenty nanograms total RNA was used to prepare libraries using Trio RNA-Seq library preparation kit (Tecan Genomics Inc.) following the manufacturer’s instructions. The protocol contains the following steps: DNase treatment to remove any DNA in the sample, first-strand and second-strand cDNA synthesis from the input RNA, single primer isothermal amplification (SPIA) of the resultant cDNAs, enzymatic fragmentation and construction of unique barcoded libraries, PCR library amplification (4 cycles were used), and an AnyDeplete step to remove rRNA transcripts. The Agencourt AMPure XP bead (Beckman Coulter) purified libraries were quantified by quantitative PCR, and the size distribution was checked using Agilent TapeStation 2200 system. The libraries were subjected to paired-end 50 bp sequencing on a NovaSeq 6000 sequencer (Illumina).

Sequencing reads were mapped to the mouse reference genome (GRCm38.85/mm10) using the STAR aligner (v2.5.0c). Alignments were guided by a Gene Transfer Format file. Mean read insert sizes and their standard deviations were calculated using Picard tools (v1.126; http://broadinstitute.github.io/picard). Read count tables were generated using HTSeq (v0.6.0), normalized based on their library size factors using DEseq2, and differential expression analysis was performed. Read per million normalized BigWig files were generated using BEDTools (v2.17.0; https://bioweb.pasteur.fr/packages/pack@bedtools@2.17.0) and bedGraphToBigWig tool (v4; https://www.encodeproject.org/software/bedgraphtobigwig/23: 1140g).

### Recombinant DNA and retroviral transduction of T cells

For overexpression of S1PR1 mutants, cDNAs (synthesized by GenScript) were cloned into the retroviral plasmid MIGR1. MIGR1 was a gift from Warren Pear (Addgene plasmid 27490). Mutations in ST10A were alanine substitutions for S336, S351, S353, S355, S358, S359, T371, S374, S375, and S380. Plat-E cells, transfected with the constructs using Lipofectamine 2000 (Thermo Fisher Scientific), were used to produce retrovirus. Before retroviral transduction, naive T cells from *S1pr1Δ* LNs were isolated by negative selection using biotinylated antibodies against CD11b, CD11c, CD19, CD25, NK1.1, TCRγΔ, and Ter119 and Magnisort Negative selection beads (Thermo Fisher Scientific) according to the manufacturer’s instructions. The cells were activated using anti-CD3ε and anti-CD28 cross-linking with 10 ng/mL IL-2. T cells were transduced with virus at 24 hours and 40 hours after activation. For transduction, cells were centrifuged at 30°C at 1,140*g* for 90 minutes in the presence of 4 μg/mL Polybrene (Sigma-Aldrich). Sixty-four hours after activation, T cells were removed from anti-CD3ε/anti-CD28 and IL-2 stimulation and changed to medium containing 5 ng/mL IL-7 and 10 ng/mL IL-15 to generate T cells with central memory properties. T cells in IL-7 and IL-15 medium were split 1:2 daily into new medium containing IL-7 and IL-15, and cultured for a total of 3 days. We did not sort cells before transfer. Instead, in analysis of recipient mice, we identified transferred cells using GFP (from the MIGR1 vector), and gated on cells with similar GFP expression for comparisons.

### Transmission electron microscopy

FACS-sorted naive CD4^+^ T cells from LNs and spleen were pelleted at 400*g* for 4 minutes by swinging bucket Eppendorf 5810 into BEEM conical capsule tips (69913-05, Electron Microscopy Sciences), and fixed in 2% glutaraldehyde and 2% paraformaldehyde in 0.1 M sodium cacodylate buffer (CB; pH 7.2) overnight at 4°C. The sample was processed following the reduced osmium-thiocarbohydrazide-osmium (rOTO) method (69, 70). Briefly, after washing with 0.1 M CB three times for 5 minutes each, the cells were postfixed with 2% osmium tetroxide and 1.5% potassium ferrocyanide in 0.1 M CB for 1.5 hours on ice. After 5 washes of 3 minutes each in ddH_2_O at room temperature, cells were placed in a filtered solution of 1% thiocarbohydrazide (Electron Microscopy Sciences) in ddH_2_O for 20 minutes at room temperature to allow additional staining. Cells were washed 5 times in ddH_2_O again on ice, and placed in 2% aqueous OsO_4_ for an additional 40 minutes on ice. Finally, cells were washed an additional 5 times in ddH_2_O on ice, and placed in 1% aqueous uranyl acetate at 4°C overnight. Cells were washed 5 times in ddH_2_O at room temperature, and en bloc lead staining was performed to enhance membrane contrast as follows. A lead aspartate solution was made by dissolving of 0.066 g of lead nitrate in 10 mL of 0.003 M aspartic acid. The pH was adjusted to 5.5 with 1N KOH, and the solution was placed in a 60°C oven for 30 minutes. The lead aspartate solution was filtered, and cells were stained at 60°C for 30 minutes, then washed 5 times in ddH_2_O at room temperature and dehydrated in a graded series of ice-cold ethanol solutions (30%, 50%, 70%, 85%, 95%, 100%, 100%; 10 minutes each). Cells were then washed in ice-cold acetone 2 times for 10 minutes each, followed by 2 acetone washes of 10 minutes each at room temperature. Cells were infiltrated gradually with EMbed812 epoxy resin (Electron Microscopy Sciences) at room temperature by placing in 50% resin/acetone for 4 hours, and 70% resin/acetone overnight. Cells were embedded in fresh 100% resin, and placed in a 60°C oven for 72 hours to allow resin polymerization. The EMbed812 resin recipe was 5 mL EMbed812, 4 mL DDSA, 2 mL NMA, 0.3 mL BDMA. Seventy-nanometer sections were cut, mounted on 200 mesh copper grids, and imaged with a Talos120C transmission electron microscope (Thermo Fisher Scientific) with a Gatan (4k × 4k) OneView Camera (Gatan Inc.).

### Mitochondrial morphological analysis

Mitochondrial numbers and cross-sectional surface area were obtained using ImageJ (NIH) by manual tracing of only clearly discernible outlines of mitochondria on transmission electron microscope images. Approximately 25–30 whole-cell images were taken per sample at the same magnification for each group within an experiment.

### Statistics

Statistical analysis was performed using GraphPad Prism v9. Graphs show mean **±** SEM. *P* values were calculated using unpaired 2-tailed Student’s *t* test, 1-way ANOVA with multiple comparisons, or Brown-Forsythe and Welch’s ANOVA with multiple-comparison test, as indicated. *P* values under 0.05 were considered significant.

### Study approval

All animal experiments were performed in accordance with protocols approved by the New York University Grossman School of Medicine Institutional Animal Care and Use Committee. Peripheral blood was drawn from anonymous adult patients and healthy controls under the protocol “Mucosal immune profiling in patients with inflammatory bowel disease,” IRB S12-01137.

### Data availability

Values for all data points in graphs are reported in the Supporting Data Values file. Requests for further information should be directed to the corresponding author. RNA-Seq data sets are in the NIH Gene Expression Omnibus database (GEO GSE221482).

## Author contributions

DD designed and performed experiments and wrote the manuscript. VMH, EYSZ, and MO performed experiments. JEC designed and performed experiments. JEA and KC provided patient samples. SRS designed experiments and edited the manuscript.

## Supplementary Material

Supplemental data

Supporting data values

## Version 1 01/09/2024

In-Press Preview

## Version 2 02/15/2024

Electronic publication
