# Supplementary material for: S1PR1 inhibition induces proapoptotic signaling in T cells and limits humoral responses within lymph nodes
Source: J Clin Invest. 2024 Jan 9;134(4):e174984. doi: 10.1172/JCI174984 (PMC10869180; doi:10.1172/JCI174984)
Supplement: Supplemental data [file jci-134-174984-s031.pdf]

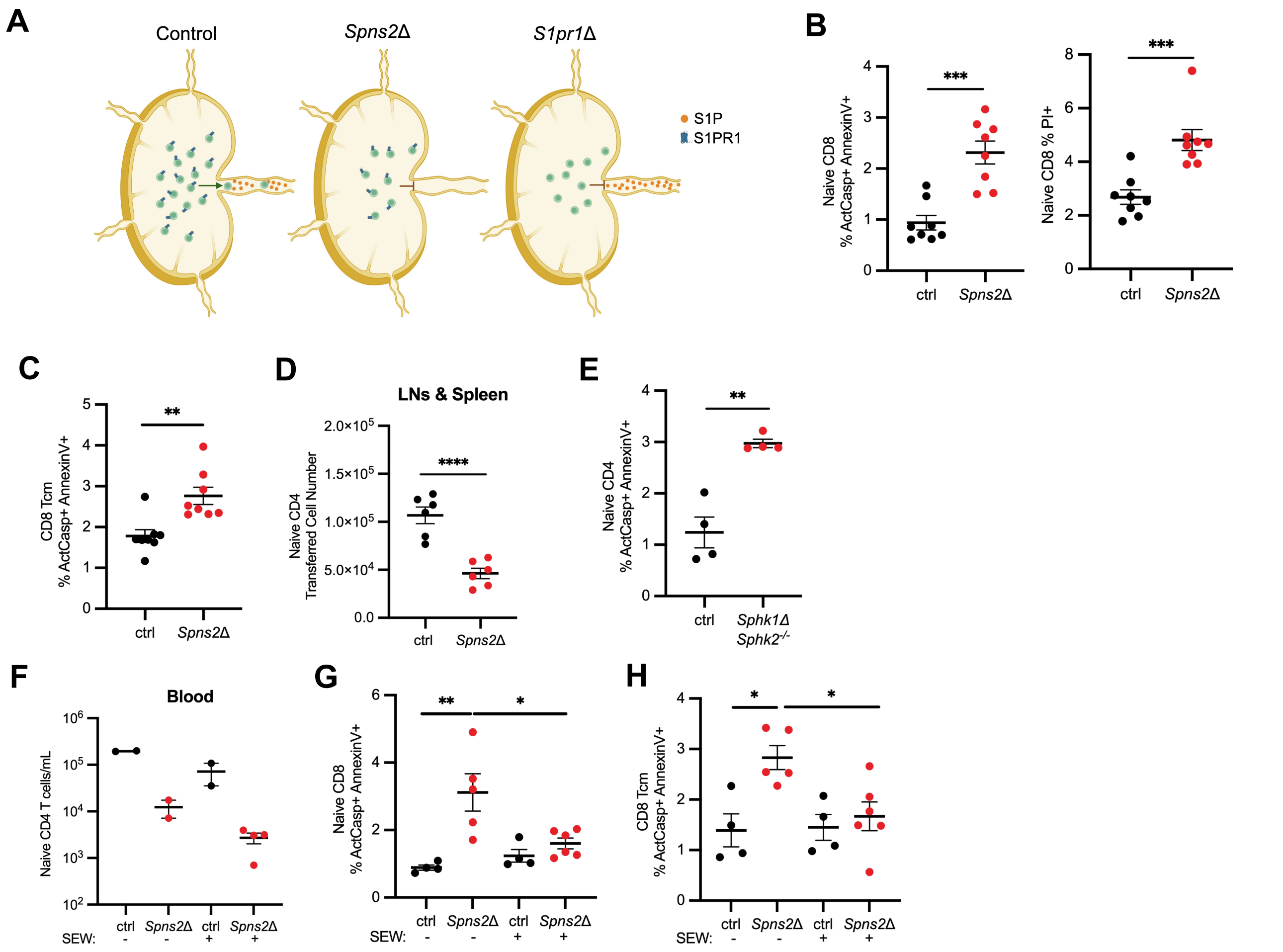

Supplementary Figure 1: SPNS2-derived S1P and S1PR1 prevent apoptotic death of naïve T cells.

A) Schematic of naïve T cell trafficking. Left: In a normal mouse, S1P is high in lymph compared to LN, and T cells express S1PR1. S1PR1 guides T cells out of the low-S1P environment of LN into the high-S1P environment of lymph. Center: In a SPNS2-deficient mouse, lymph S1P is lost. T cells still express S1PR1, but without lymph S1P to attract T cells out of the LN, T cells do not exit. Right: In an S1PR1-deficient mouse, S1P remains high in lymph compared to LN. However, without S1PR1, T cells cannot sense the S1P gradient and do not exit. Created with biorender.com.

B-C) *Spns2*$^{f/f}$ *UBC*-CreERT2 mice and littermate controls were treated with tamoxifen, and 3-4 weeks later LN T cells were analyzed by flow cytometry. (B) Frequency of ActCasp+ AnnexinV+ and PI+ cells among naïve CD8+ T cells. (C) Frequency of ActCasp+ AnnexinV+ among CD8+ Tcm (CD62L+CD44hi). Compilation of 4 experiments with n=8 mice for both groups.

D) *Spns2*$^{f/f}$ *UBC*-CreERT2 mice and littermate controls were treated with tamoxifen. 3-4 weeks after tamoxifen treatment, the mice received $1x10^6$ CD45.1+ lymphocytes intravenously. Three weeks post-transfer, naïve CD4+ CD45.1+ T cells remaining in the recipient animals were enumerated (total reflects sum of cells in skin-draining LN, mesenteric LN, and spleen). Compilation of 3 experiments with n=6 mice per group.

E) Frequency of ActCasp+ AnnexinV+ cells among naïve CD4+ T cells in LN of *Sphk1*$^{f/f}$*Sphk2*$^{-/-}$ *Lyve1*-Cre mice and littermate controls. Compilation of 2 experiments with n=4 mice for both groups.

F-H) *Spns2*$^{f/f}$ *Lyve1*-Cre mice and littermate controls were treated with 10mg/kg SEW-2871 or vehicle daily. After ten days of treatment, T cells were analyzed by flow cytometry. (E) Number of naïve CD4+ T cells in the blood of *Spns2*Δ mice and littermate controls, with and without SEW-2871 treatment. Frequency of ActCasp+ AnnexinV+ cells among (G) naïve CD8+ T cells and (H) CD8+ Tcm (CD62L+CD44hi) in LN of *Spns2*Δ mice and littermate controls, with and without SEW-2871 treatment. Compilation of 3 experiments with n=3-4 mice per group.

Statistical analysis: Student's t test was performed for panels B-E. One-way ANOVA was performed for panel G-H. *p ≤ 0.05, **p ≤ 0.01, ***p ≤ 0.001, ****p ≤ 0.0001, N.S. non-significant.

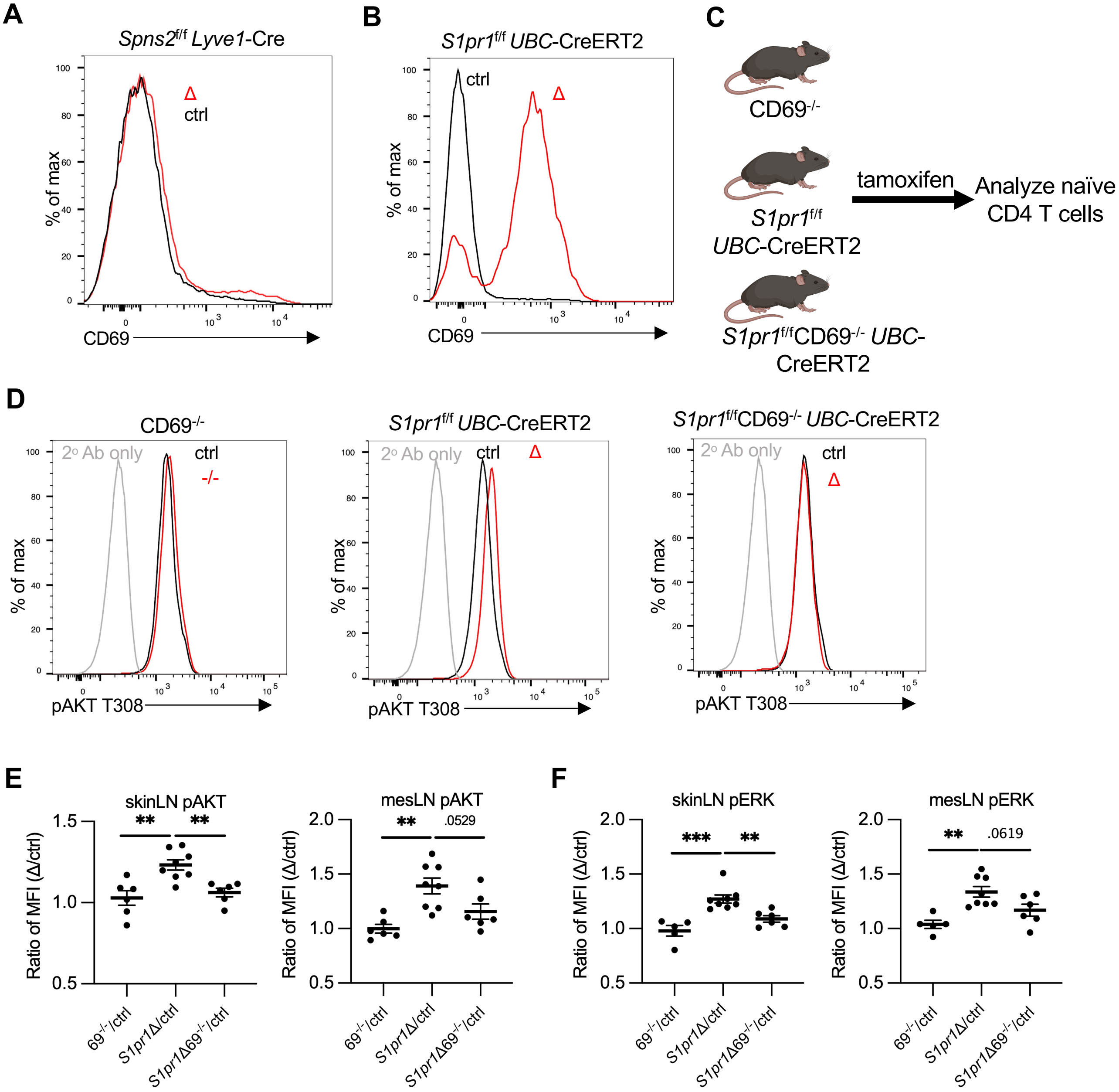

Supplementary Figure 2: CD69 regulates increased pAKT and pERK in S1PR1-deficient naïve T cells.

Text: One distinction between naïve T cells from *Spns2*Δ and *S1pr1*Δ mice is that naive *S1pr1*Δ T cells have high surface levels of the C-type lectin CD69 compared to controls, while naïve T cells from *Spns2*Δ mice do not have detectable surface CD69 (Figures S2A-S2B). CD69 and S1PR1 play antagonistic roles on the cell surface (71). Naïve T cells express high levels of S1PR1, which binds basal CD69 and maintains its low surface expression. A recently activated T cell robustly increases *Cd69* transcription, which shifts the balance; the increased CD69 binds S1PR1 and forces its internalization, keeping T cells at the site of activation. Naïve *S1pr1*Δ T cells have high levels of surface CD69 at baseline. Much remains to be learned about CD69's function, but we hypothesized that surface CD69 might promote AKT and ERK signaling, as these pathways are normally induced early during T cell activation. To test this, we crossed *S1pr1*$^{f/f}$*UBC*-CreERT2 with *Cd69*$^{-/-}$ mice(64) and asked whether loss of CD69 reduced AKT and ERK phosphorylation in *S1pr1*Δ T cells (Figure S2C). *S1pr1*Δ naïve T cells had increased expression of pAKT and pERK compared to littermate controls and *Cd69*$^{-/-}$ T cells, but *S1pr1*Δ; *Cd69*$^{-/-}$ naïve T cells had similar expression of pAKT and pERK to controls and *Cd69*$^{-/-}$ T cells (Figures S2D-S2F). This suggests that high expression of CD69 in *S1pr1*Δ naïve T cells partially explains the increased pAKT and pERK.

Legend: A-B) Representative histogram of CD69 surface expression on naïve CD4+ T cells from LN of (A) *Spns2*Δ and (B) *S1pr1*Δ (red) or littermate control (black) mice.

C-F) *S1pr1*$^{f/f}$ *UBC*-CreERT2 mice, CD69$^{-/-}$ mice, or *S1pr1*$^{f/f}$ *UBC*-CreERT2; CD69$^{-/-}$ mice and their respective littermate controls were treated with tamoxifen. 3-4 weeks later, T cells from either skin-draining LN or mesenteric LN were analyzed by flow cytometry for pAKT and pERK expression. (C) Experiment diagram. (D) Representative histograms of pAKT expression by naïve CD4 T cells from skin-draining LN of the indicated mice. (E-F) Compilation. Each point represents ratio of (E) pAKT or (F) pERK geometric MFI in naïve CD4+ T cells between a *S1pr1*Δ, CD69$^{-/-}$, or *S1pr1*Δ CD69$^{-/-}$ mouse and its respective littermate control. Compilation of 6 experiments with n=6-8 mice per group.

Statistical analysis was performed using One-way ANOVA with multiple comparisons. *$p \leq 0.05$, **$p \leq 0.01$, ***$p \leq 0.001$, ****$p \leq 0.0001$, N.S. non-significant.

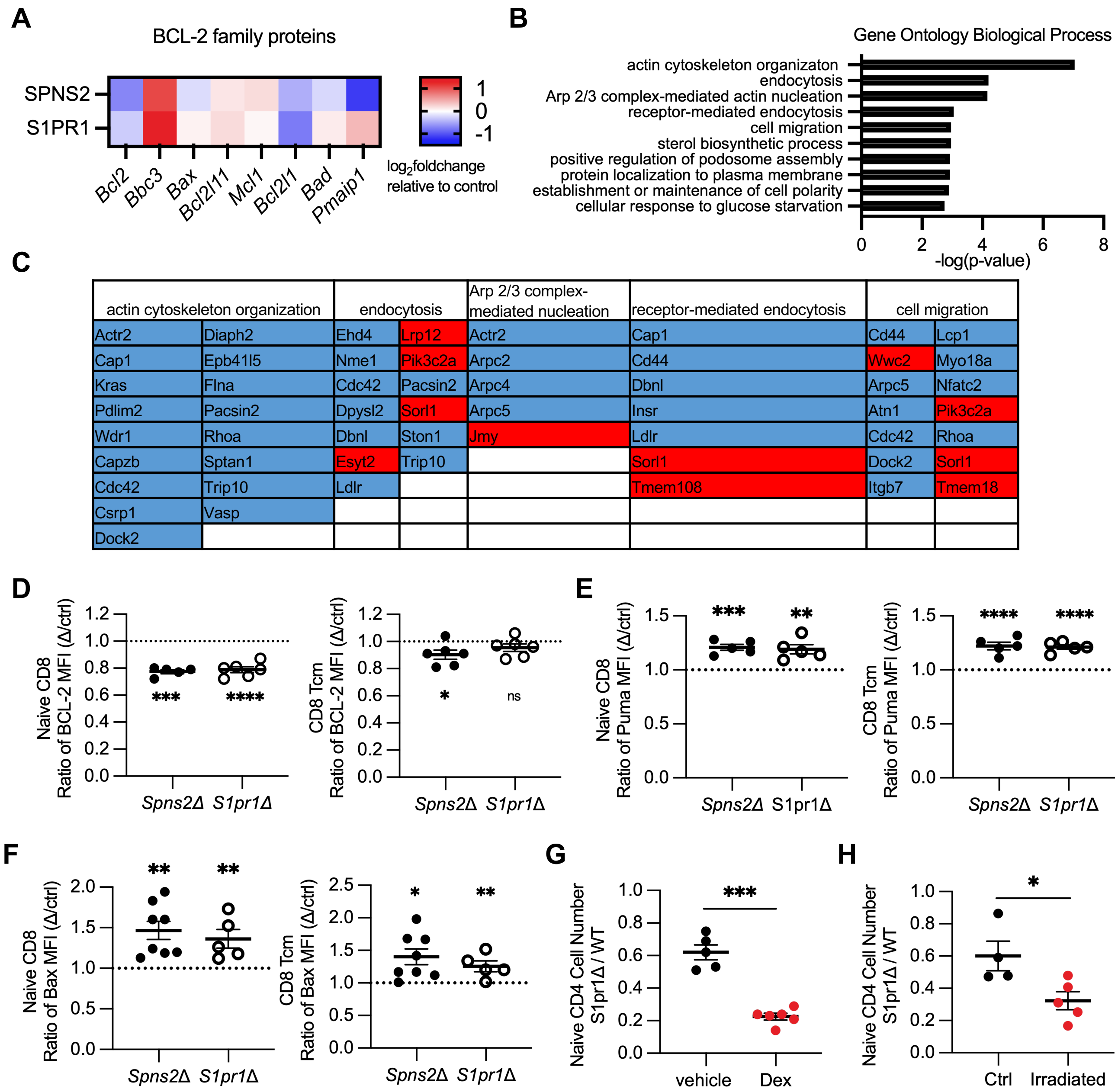

Supplementary Figure 3: Expression of BCL-2 family proteins in the absence of S1P signaling.
A-C) For SPNS2: CD45.2 *Spns2*[f/f] *Lyve1*-Cre mice or CD45.2 littermate control mice were lethally irradiated and reconstituted with WT CD45.1/.2 bone marrow. After reconstitution, naïve CD4 T cells were isolated and RNA-Seq was performed. For S1PR1: a 50:50 mixture of CD45.2 UBC-GFP: CD45.2 *S1pr1*[f/f] *UBC*-CreERT2 or CD45.2 *UBC*-GFP: CD45.2 *S1pr1*[f/f] control bone marrow was transferred into lethally irradiated CD45.1/.2 WT mice. After reconstitution, mice were treated with tamoxifen. Four weeks after tamoxifen treatment, naïve T cells were isolated and RNA-Seq was performed. (A) Heatmap of BCL-2 family proteins in naïve CD4 T cells from *Spns2*Δ and *S1pr1*Δ mice. Heatmap represents log_2fold change of genes from either *Spns2*Δ mice or *S1pr1*Δ mice relative to control cells. (B) Differentially expressed genes shared between both *Spns2*Δ and *S1pr1*Δ datasets were used to generate top 10 Gene Ontology (GO) biological process using DAVID Bioinformatics Resource. (C) Differentially expressed genes shared between both *Spns2*Δ and *S1pr1*Δ datasets listed under top Gene Ontology Biological Processes. Red: up in the absence of S1P signaling. Blue: down in the absence of S1P signaling.
D-F) Expression of BCL-2 family members in naïve CD8+ T cells and CD8+ Tcm (CD62L+CD44hi) from LN of *Spns2*Δ or *S1pr1*Δ mice and littermate controls. (D) Compilation of BCL-2 expression. Each point represents the ratio of BCL-2 geometric MFI between a *Spns2*Δ or *S1pr1*Δ mouse and its littermate control. Compilation of 5-6 experiments with n=5-7 pairs per group. (E) As in (D) for PUMA. Compilation of 4-5 experiments with n=4-5 pairs per group. (F) As in (D) for BAX. Compilation of 6 experiments with n=5-8 pairs per group.
G-H) *S1pr1*Δ and littermate control lymphocytes were co-transferred (1:1 by naïve CD4 T cell counts) intravenously into WT recipients. The transferred cells were labeled with CellTrace Violet or CellTrace Yellow, and dyes were swapped between experiments. 24 hours later, recipients were treated with either 2 mg/kg dexamethasone or 1 gy irradiation. 24 hours later, dye-labeled naïve CD4 T cells in LN were enumerated. Compilation of ratio of number of naïve CD4+ T cells from *S1pr1*Δ mice vs its littermate control recovered in recipients. (G) 2 mg/kg dexamethasone treatment. Compilation of 3 experiments with n=5-7 mice per group. (H) 1 gy irradiation. Compilation of 2 experiments with n=4-5 mice per group.
Statistical analysis was performed using Student's t test. *$p \leq 0.05$, **$p \leq 0.01$, ***$p \leq 0.001$, ****$p \leq 0.0001$, N.S. non-significant.

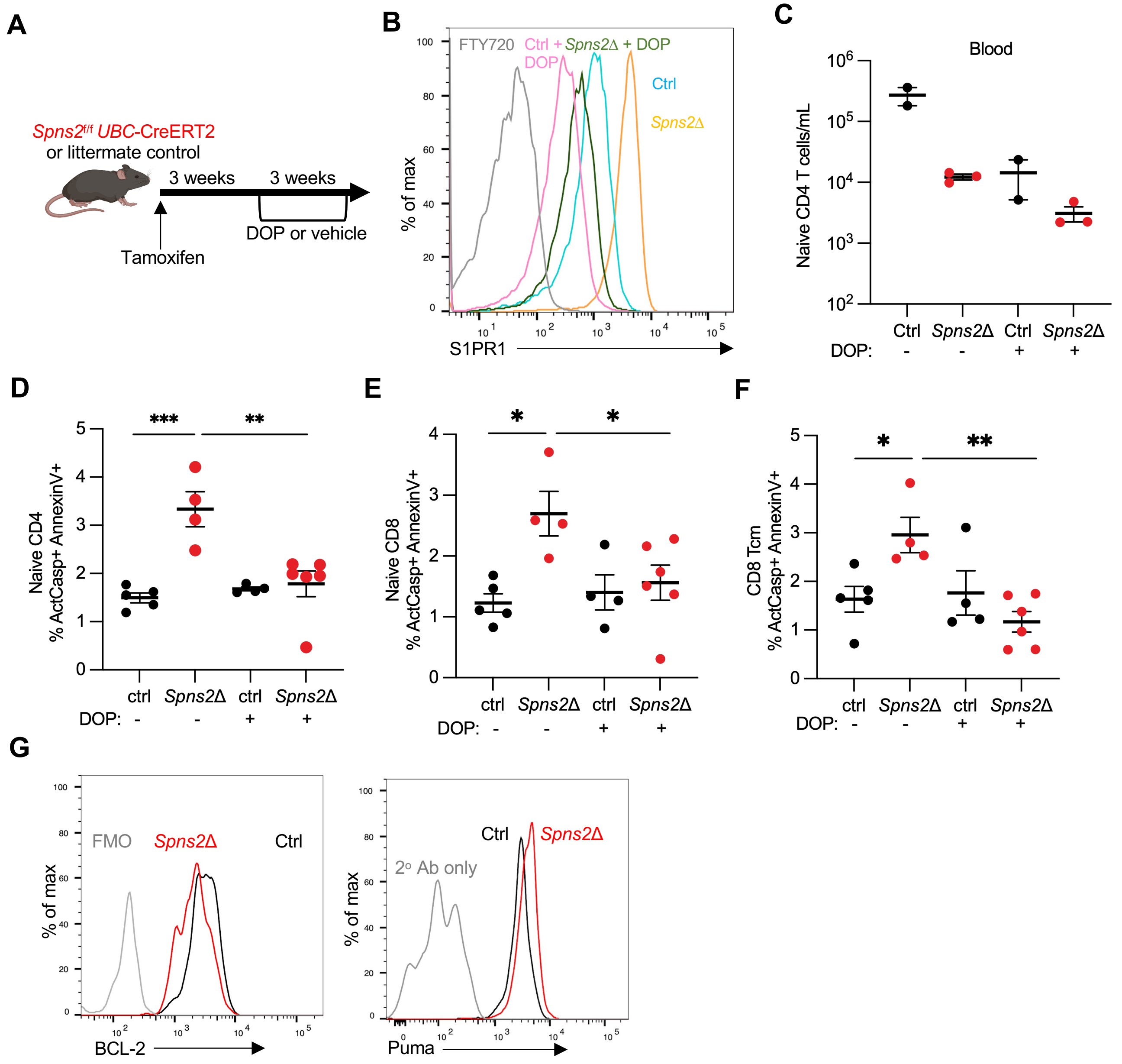

Supplementary Figure 4: BCL-2 family proteins regulate S1PR1-dependent naïve T cell survival.

A-F) *Spns2*f/f *UBC*-CreERT2 mice and littermate controls were treated with tamoxifen. At least 3 weeks later, mice were treated with 30mg/l DOP and 10mg/l sucrose, or sucrose alone, in the drinking water. After 3 weeks of DOP treatment, LN T cells were analyzed by flow cytometry. (A) Experiment design. (B) Representative histogram of surface S1PR1 expression on naïve CD4+ T cells from LN of *Spns2*Δ mice and littermate controls, with and without DOP treatment. (C) Number of naïve CD4+ T cells in the blood of *Spns2*Δ mice and littermate controls, with and without DOP treatment. Compilation of 2 experiments with n=2-3 per group. (D) Frequency of ActCasp+ AnnexinV+ cells among naïve CD4+ T cells in LN of *Spns2*Δ mice or littermate controls, with or without DOP treatment. Compilation of 3 experiments with n=4-6 mice per group. Frequency of ActCasp+ AnnexinV+ cells among (E) naïve CD8+ T cells and (F) CD8+ Tcm (CD62L+CD44hi) in LN of *Spns2*Δ mice or littermate controls, with and without DOP treatment. Compilation of 3 experiments with n=4-6 per group.

G) Representative histograms of BCL-2 and PUMA expression in *Bax*-/- naïve CD4+ T cells transferred to *Spns2*f/f *Lyve1*-Cre mice or littermate controls.

Statistical analysis was performed using one-way ANOVA was multiple comparisons. *p ≤ 0.05, **p ≤ 0.01, ***p ≤ 0.001, ****p ≤ 0.0001, N.S. non-significant.

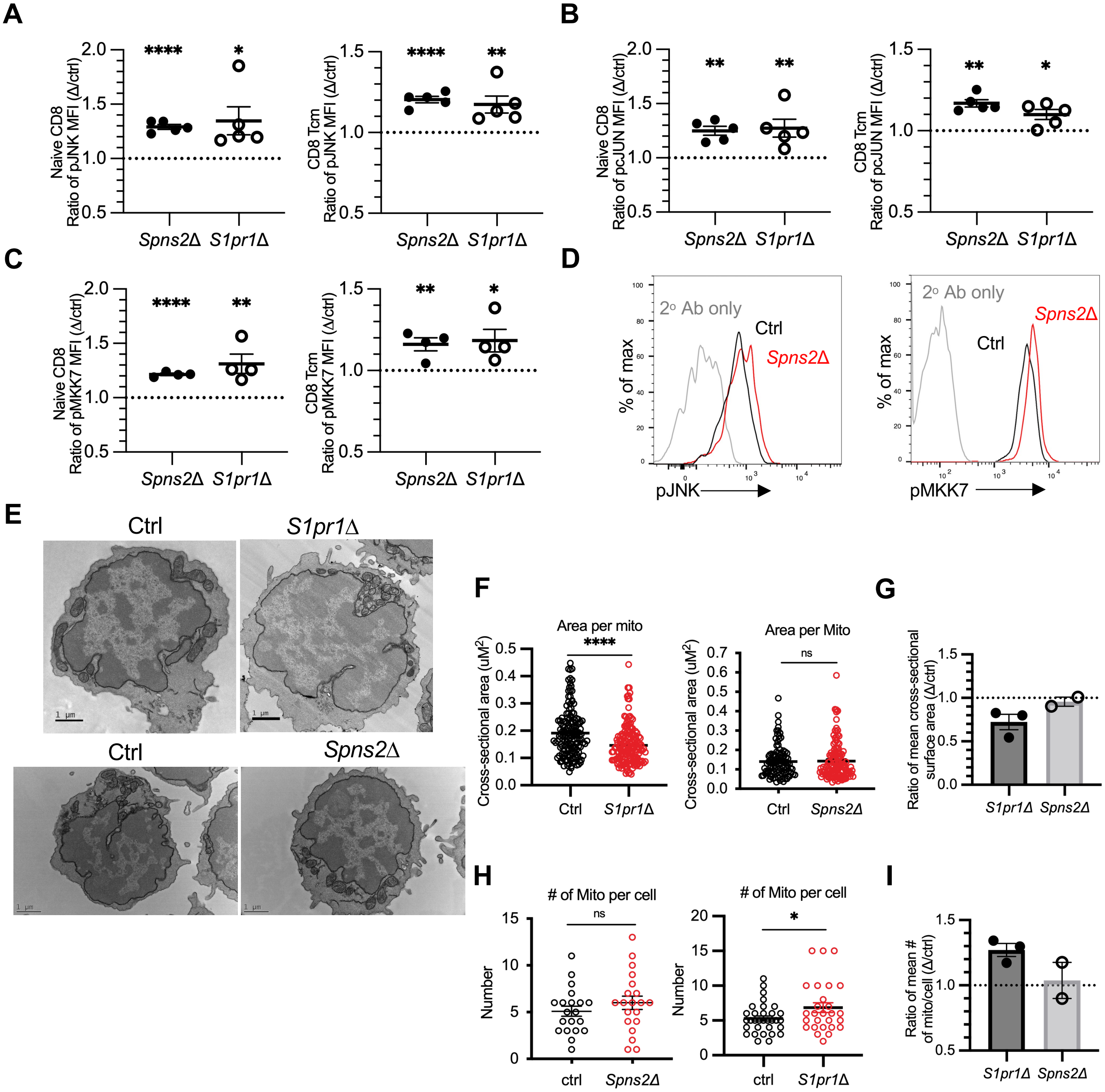

Supplementary Figure 5: JNK signaling regulates S1P-dependent naïve T cell survival.

A-C) Expression of phosphorylated JNK pathway proteins in naïve CD8+ T cells and CD8+ Tcm (CD62L+CD44hi) from LN of *Spns2*Δ or *S1pr1*Δ mice and littermate controls, analyzed by flow cytometry. (A) Compilation of relative MFI values for pJNK. Relative values represent expression in one individual mouse divided by the mean value of littermate control mice in that experiment. Compilation of 4-5 experiments with n=5 pairs per group. (B) As in (A) for pcJUN. Compilation of 4-5 experiments with n=4-5 pairs per group. (C) As in (A) for pMKK7. Compilation of 4 experiments with n=4 pairs per group.

D) Representative histograms of pJNK and pMKK7 expression in $Bax^{-/-}$ naïve CD4+ T cells transferred to $Spns2^{f/f}$ Lyve1-Cre mice or littermate controls.

E-I) Electron microscopy analysis of mitochondria in naïve CD4+ T cells sorted from LN. (E) Representative images of mitochondria within naïve CD4+ T cells from LN of a *S1pr1*Δ mouse and its littermate control or a *Spns2*Δ^{Lyve1} mouse and its littermate control. (F) Cross-sectional area of individual mitochondria, from one representative set of mice examining at least 20 cells per genotype. (G) Compilation of the ratio of the mean cross-sectional area of mitochondria in naïve CD4+ T cells of *S1pr1*Δ mice vs their littermate controls (3 pairs in 3 experiments) or of *Spns2*Δ mice vs their littermate controls (2 pairs in 2 experiments). (H) Number of mitochondria per cell, from one representative set of mice examining at least 20 cells per genotype. (I) Compilation of the ratio of the mean number of mitochondria per cell of *S1pr1*Δ mice vs their littermate controls (3 pairs in 3 experiments), or of *Spns2*Δ mice vs their littermate controls (2 pairs in 2 experiments).

Statistical analysis was performed using Student's t test. *p ≤ 0.05, **p ≤ 0.01, ***p ≤ 0.001, ****p ≤ 0.0001, N.S. non-significant.

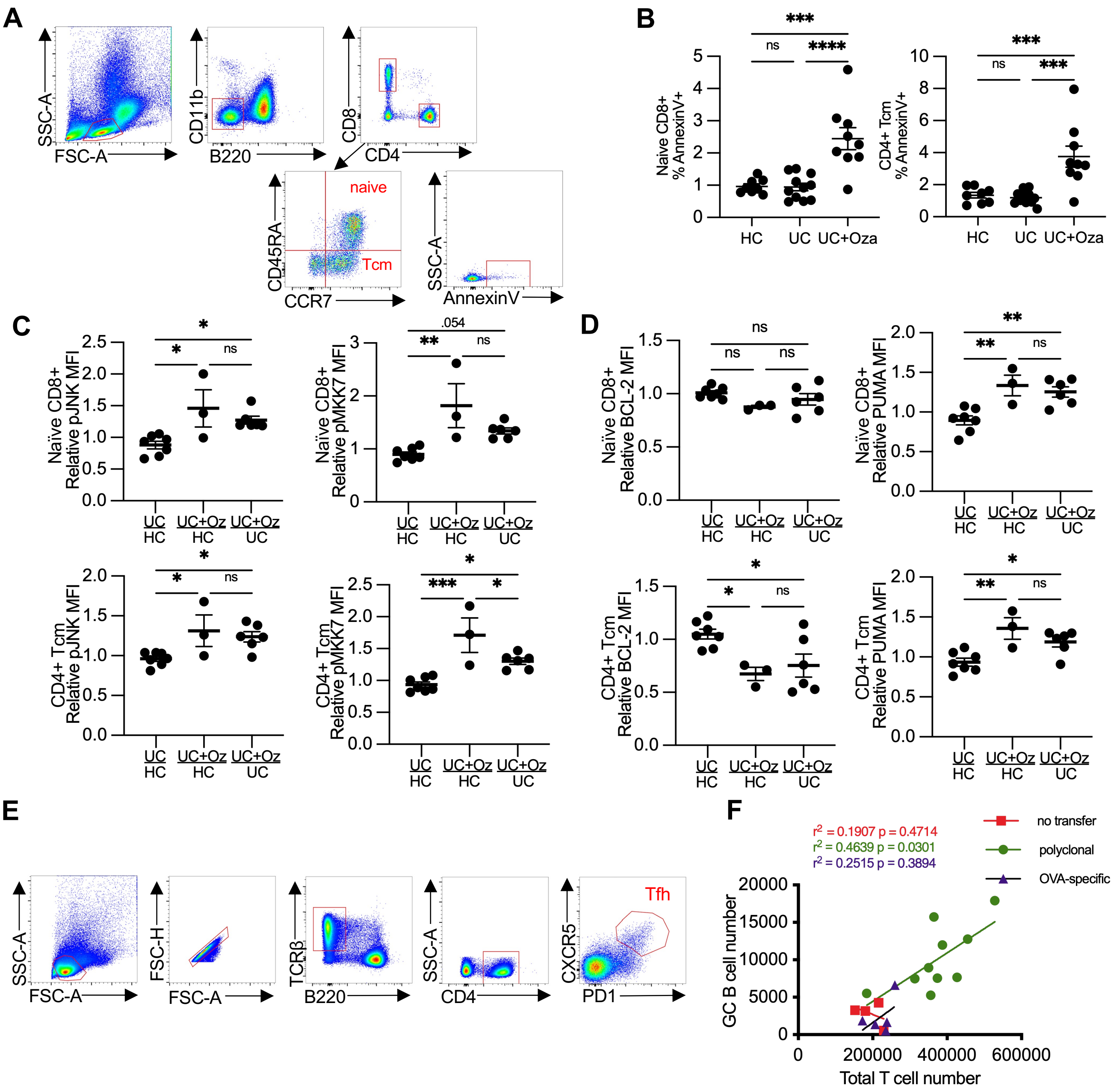

Supplementary Figure 6: S1PR1 modulators impair T cell survival in humans and germinal center responses in mice.
A-D) PBMCs were isolated from blood samples of UC patients treated with ozanimod (UC+Oza), UC patients not treated with ozanimod (UC), or healthy controls (HC), and analyzed by flow cytometry. (A) Gating strategy to analyze naïve CD4+, naïve CD8+, and CD4+ Tcm. (B) Frequency of AnnexinV+ cells among naïve CD8+ T cells or among CD4+ Tcm cells. Compilation of 10 experiments with n=8-11 per group. (C) Compilations of pJNK and pMKK7 relative geometric MFI values from naïve CD8+ T cells or from CD4+ Tcm cells, calculated as in Fig. 7F. Compilation of 10 experiments with n=3-7 per group. (D) Compilations of relative MFI values for BCL-2 and PUMA from naïve CD8+ T cells or from CD4+ Tcm cells. Compilation of 10 experiments with n=3-7 per group.
E) Gating strategy to identify Tfh cells.
F) Correlation between total T cell numbers and GC B cell numbers from ‘Long FTY + no transfer’ group, ‘Long FTY + polyclonal T cells’ group, and ‘Long FTY + OVA-specific T cells’ group.
Statistical analysis was performed using one-way ANOVA with multiple comparisons for panels B-D. *p ≤ 0.05, **p ≤ 0.01, ***p ≤ 0.001, ****p ≤ 0.0001, N.S. non-significant.

Supplementary Table 1: Characteristics of ulcerative colitis patients and healthy controls

	Healthy Controls (n=8)	UC+ No Ozanimod (n=11)	UC + Ozanimod (n=9)
Sex			
Female	5 (62.5%)	2 (18.2%)	4 (42.9%)
Male	3 (37.5%)	9 (81.8%)	5 (57.1%)
Race			
White	6 (75%)	6 (54.5%)	5 (55.6%)
Black	0	0	3 (33.3%)
Asian	0	2 (18.2%)	1 (11.1%)
Other/mixed	2 (25%)	3 (27.3%)	0
Hispanic ethnicity	0	1 (9.1%)	0
Age at Encounter (years; median, IQR)	32 (28)	33 (17)	29 (7.5)
Age at IBD diagnosis (years; median, IQR)	-	26 (15)	24 (7)
Duration of disease (years; median, IQR)		6 (10)	4 (4)
Maximum ulcerative colitis extent	-		
Proctitis		1 (9.1%)	2 (22.2%)
Left-sided		0	3 (33.3%)
Extensive colitis		9 (81.8%)	4 (42.9%)
Inflammatory biomarkers within 30 days	-		
C-rp (median, IQR)		0.7 (2.7)	1.3 (4.6)
Fecal calpro (median, IQR)		262 (250)	839 (435)
Mayo endoscopic subscore within 30 days	-		
0		4 (36.3%)	1
1		2 (18.2%)	0
2		2 (18.2%)	4 (42.9%)
3		1 (9.1%)	2 (22.2%)
Not assessed		2 (18.2%)	2 (22.2%)
IBD therapy exposure history	-		
Mesalamines		10 (90.9%)	9 (100%)
Corticosteroids		9 (81.8%)	8 (88.9%)
Immunomodulators		2 (18.2%)	2 (22.2%)
Anti-TNF		4 (36.3%)	2 (22.2%)
Vedolizumab		1 (9.1%)	2 (22.2%)
JAK inhibitor		0	2 (22.2%)

Supplementary Table 2: Materials used in the study

Reagent or Resource	Source	Identifier
Antibodies		
APC-conjugated Streptavidin	BioLegend	catalog no. 405207
APC-conjugated anti-mouse CD44 (IM7)	BioLegend	catalog no. 103012 RRID:AB_312963
APC-conjugated anti-human CCR7 (G043H7)	BioLegend	catalog no. 353213 RRID:AB_10915474
APC-conjugated anti-mouse CD45.2 (104)	BioLegend	catalog no. 109814 RRID: AB_389211
APC-Cy7-conjugated anti-human CD11b (MI/70)	BioLegend	catalog no. 101226 RRID: AB_830642
APC-e780-conjugated anti-mouse CD8a (5.3-6.7)	Thermo Fisher Scientific	catalog no. 47-0081-82 RRID: AB_1272185
APC-e780 conjugated anti-TCR-beta (H57-597)	Thermo Fisher Scientific	catalog no. 47-5961-82 RRID: AB_1272173
Biotinylated Affinipure Fragment Donkey anti-Rat	Jackson Immunoresearch	catalog no. 712-066-153 RRID: AB_2340649
BUV395-conjugated anti-mouse CD4 (GK1.5)	BD Biosciences	catalog no. 740208 RRID: AB_2734761
BUV395- conjugated anti- B220 (RA3-6B2)	BD Biosciences	catalog no.563793 RRID: AB_2738427
BV421-conjugated anti-mouse CD62L (MEL-14)	BioLegend	catalog no. 104436 RRID:AB_2562560
BV421-conjugated anti-human CD45RA (HI100)	BioLegend	catalog no. 304130 RRID:AB_10965547
BV510-conjugated anti-mouse CD62L (MEL-14)	BioLegend	catalog no. 104441 RRID: AB_2561537
BV650-conjugated anti-mouse CD25 (PC61)	BioLegend	catalog no. 102038 RRID: AB_2563060
FITC-conjugated anti-mouse CD45.1 (A20)	BioLegend	catalog no. 110706 RRID: AB_313495
FITC-conjugated anti-mouse CD62L (MEL-14)	BioLegend	catalog no. 104408 RRID: AB_313095
PE-conjugated anti-mouse CD69 (H1.2F3)	BioLegend	catalog no. 104508 RRID: AB_313111
PE-conjugated anti-mouse CD44 (IM7)	BioLegend	catalog no. 103024 RRID:AB_493687
PE-conjugated anti-mouse CD45.1 (A20)	BioLegend	catalog no. 110708 RRID:AB_313496

PE-conjugated anti-mouse CD127 (A7R34)	BioLegend	catalog no.135010 RRID: AB_1937251
PE-conjugated anti-human CCR7 (G043H7)	BioLegend	catalog no. 353203 RRID:AB_10916391
PE-conjugated anti-mouse BCL-2 (BCL/10C4)	BioLegend	catalog no. 633508 RRID: AB_2290367
PE-conjugated anti-human BCL-2 (100)	BioLegend	catalog no. 658707 RRID:AB_2563282
PerCP-Cy5.5-conjugated anti-mouse B220 (RA3-6B2)	BioLegend	catalog no. 103236 RRID: AB_893354
PerCP-Cy5.5-conjugated anti-mouse CD44 (IM7)	BioLegend	catalog no. 103032 RRID: AB_2076204
PerCP-Cy5.5-conjugated anti-mouse CD45.2 (104)	BioLegend	catalog no. 109828 RRID:AB_893350
Rat anti-mouse S1PR1 (713412)	R&D Systems	catalog no. MAB7089
Rabbit anti-phospho AKT T308 (D25E6)	Cell Signaling Technology	catalog no. 13038S
Rabbit anti-phospho-p44/p42 ERK1/2 Thr 202/Tyr 204	Cell Signaling Technology	catalog no. 9101L
Rabbit anti-phospho-S6 Ribosomal protein (Ser235/236)	Cell Signaling Technology	catalog no. 2211L
Rabbit polyclonal anti-PUMA	ProSci	catalog no. 3043
Mouse anti-Bax (6A7)	Thermo Fisher Scientific	catalog no. MA5-14003 RRID: AB_10979735
Rabbit anti-phospho-JNK1/2 (D12H7L17)	Thermo Fisher Scientific	catalog no. 700031 RRID: AB_2532273
Mouse anti-phospho-cJUN (KM-1)	Santa Cruz Biotechnology	catalog no. sc-822
Rabbit anti-phospho-MKK7 (Ser271,Thr 275) (MKK7S271T275-R4F9)	Thermo Fisher Scientific	catalog no. MA5-28042
Rabbit anti-phospho-CREB (87G3)	Cell Signaling Technology	catalog no. 9198S
Alexa Fluor 647-conjugated donkey anti-rabbit IgG	Jackson Immunoresearch	catalog no. 711-605-152 RRID:AB_2492288
Alexa Fluor 647-conjugated donkey anti-mouse IgG	Jackson Immunoresearch	catalog no. 715-605-150 RRID: AB_2340862
Alexa Flour 647-conjugated anti-mouse BCL-2 (BCL/10C4)	BioLegend	catalog no. 633510 RRID:AB_2274702
Biotinylated anti-mouse CD8a (5.3-6.7)	BioLegend	catalog no. 100704 RRID: AB_312743
Biotinylated anti-mouse CD11c (N418)	BioLegend	catalog no. 117304 RRID: AB_313773

Biotinylated anti-mouse CD11b (M1/70)	BioLegend	catalog no. 101204 RRID: AB_312787
Biotinylated anti-mouse NK1.1 (PK136)	BioLegend	catalog no. 108704 RRID: AB_313391
Biotinylated anti-mouse Ter119 (TER-119)	BioLegend	catalog no. 116204 RRID:AB_313705
Biotinylated anti-mouse CD19 (6D5)	BioLegend	catalog no. 115504 RRID:AB_313639
Biotinylated anti-mouse B220 (RA3-6B2)	BioLegend	catalog no. 103204 RRID:AB_312989
Biotinylated anti-mouse CD25 (PC61)	BioLegend	catalog no. 102004 RRID:AB_312853
Biotinylated anti-mouse TCR γ/δ (GL3)	BioLegend	catalog no. 118103 RRID:AB_313827
Biotinylated anti-mouse CD44 (IM7)	BioLegend	catalog no. 103003 RRID:AB_312954
InVivoMab anti-mouse CD3ε (145-2C11)	Bio X Cell	catalog no. BE0001-1 RRID:AB_1107634
InVivoMab anti-mouse CD28 (37.51)	Bio X Cell	catalog no. BE0015-1 RRID:AB_1107628
InVivoMab rat IgG2A isotype control (2A3)	Bio X Cell	catalog no. BE0089 RRID:AB_1107769
InVivoMab anti-mouse CD127 (A7R34)	Bio X Cell	catalog no. BE0065 RRID:AB_1107590
Chemicals, Peptides, and Recombinant Proteins		
PBS	Gibco	catalog no. 14190250
RPMI-1640	Gibco	catalog no. 11875119
DMEM	Corning	catalog no. 10014CV
OptiMEM	Gibco	catalog no. 51985034
Tamoxifen	Sigma-Aldrich	catalog no. T5648
ABT-199	Tocris	catalog no. 6960
SP600125	Selleckchem	catalog no. S1460
H-89	Selleckchem	catalog no. S1582
JNK-IN-8	MedChemExpress	catalog no. HY-13319
Dexamethasone	Sigma-Aldrich	catalog no. D1756
SEW-2871	Cayman Chemical	catalog no. 10006440
Fingolimod	Cayman Chemical	catalog no. 10006292
4-deoxypyridoxine-HCl	Sigma-Aldrich	catalog no. D0501
Lipofectamine 2000	Thermo Fisher	catalog no. 11668027
Polybrene	Sigma-Aldrich	catalog no. TR-1003
Lymphoprep	Stem Cell Technologies	catalog no. 7801
Recombinant murine IL-7	Peprotech	catalog no. 217-17

Recombinant human IL-2	Peprotech	catalog no. 200-02
Recombinant murine IL-15	Peprotech	catalog no. 210-15
Fas ligand	Enzo Life Sciences	catalog no. ALX-850-014-KI02
TRIzol LS	Thermo Fisher Scientific	catalog no. 10296010
Linear Acrylamide	Thermo Fisher Scientific	catalog no. am9520
Magnisort Negative Selection Beads	Thermo Fisher Scientific	catalog no. MSNB-6002-71
FITC-conjugated caspACE-VAD-FMK	Promega	catalog no. G7461
Pacific Blue-conjugated AnnexinV	BioLegend	catalog no. 640918
APC-conjugated AnnexinV	BioLegend	catalog no. 640920
AnnexinV Binding Buffer	BioLegend	catalog no. 422201
Corn Oil	Sigma-Aldrich	catalog no. C8267
Sucrose	Sigma-Aldrich	catalog no. S9378
Tween 20	Sigma-Aldrich	catalog no. P1379
Tween 80	Sigma-Aldrich	catalog no. P1754
PEG400	Sigma-Aldrich	catalog no. PX1286B-2
Critical Commercial Assays		
Rneasy Plus Micro Kits	QIAGEN	catalog no. 74034
Rnase-free Dnase set	QIAGEN	catalog no. 79254
eBioscience FoxP3 staining kit	Thermo Fisher Scientific	catalog no. 00-5523-00
Cytofix/Cytoperm Kit	BD Biosciences	catalog no. 554714
CellTrace Yellow Cell Proliferation Kit for Flow Cytometry	Thermo Fisher Scientific	catalog no. C34567
CellTrace Violet Cell Proliferation Kit for Flow Cytometry	Thermo Fisher Scientific	catalog no. C34557
Live/DEAD Fixable Blue Dead Cell Stain Kit	Thermo Fisher Scientific	catalog no. L23105
Experimental Models: Cells/ Cell Lines		
Grk2$^{f/-}$ *Cd4*-Cre and Grk2$^{f/+}$ Cd4-Cre bone marrow	Laboratory of Jason Cyster	Arnon et al., 2011
Platinum-E (plat-E) cell line	Laboratory of T Kitamura	Morita et al., 2000
Sheep Red Blood Cells	Colorado Serum Company	catalog no. 31112
Experimental Models: Organisms/ Strains		
C57BL/6J	Jackson Laboratory	RRID: IMSR_JAX:000664
B6.SJL-*Ptprc*^{a}*Pepc*^{b}/BoyJ	Jackson Laboratory	RRID: IMSR_JAX:002014
Spns2$^{f/f}$	This laboratory	Mendoza et al., 2012
S1pr1$^{f/f}$	Jackson laboratory	RRID: IMSR_JAX:019141
UBC-CreERT2	Jackson laboratory	RRID: IMSR_JAX:007001
Lyve1-Cre	Laboratory of Jason Cyster	Pham et al., 2010
Bax$^{-/-}$	Jackson Laboratory	RRID: IMSR_JAX:002994

MHCII$^{-/-}$	Jackson Laboratory	RRID: IMSR_JAX:003584
Cd69$^{-/-}$	Laboratory of Jason Cyster	Murata et al., 2003
Sphk1$^{f/f}$	Laboratory of Jason Cyster	Pappu et al., 2007
Sphk2$^{-/-}$	Jackson Laboratory	RRID: IMSR_JAX:019140
OT-I: C57BL/6-Tg(TcraTcrb)1100Mjb/J	Jackson Laboratory	IMSR_JAX:003831
OT-II: B6.Cg-Tg(TcraTcrb)425Cbn/J	Jackson Laboratory	IMSR_JAX:004194
Recombinant DNA		
pMIGR1	Addgene (gift from Warren Pear)	RRID: Addgene_27490
WT S1PR1-pMIGR1	This laboratory	
ST10A S1PR1-pMIGR1	This laboratory	
Software and algorithms		
GraphPad Prism 9.0	GraphPad	https://www.graphpad.com/scientific-software/prism/
FlowJo v 10	TreeStar	https://www.flowjo.com/solutions/flowjo
FACSDiva v8.02	BD Biosciences	https://www.bdbiosciences.com/en-us/instruments/research-instruments/research-software/flow-cytometry-acquisition/facsdiva-software
Biorender	Biorender	www.biorender.com
ImageJ v 1.49	NIH	https://imagej-nih-gov.ezproxy.med.nyu.edu/ij/
Other		
LSRII Flow Cytometer	BD Biosciences	N/A
Beckman Coulter Multisizer 3	Beckman Coulter	catalog no. 383601
LightCycler 480 System	Roche Diagnostics	catalog no. 5015243001
